# A Redox‐Active and Electroactive Hydrogel Enabled by an Integrated PEDOT@PMOF Nanofiller for Post‐Infarct Myocardial Repair

**DOI:** 10.1002/advs.202505612

**Published:** 2025-09-12

**Authors:** Shuyi He, Linyu Long, Wenqi Liu, Zhicun Wang, Xiaofeng Wu, Li Yang, Yunbing Wang

**Affiliations:** ^1^ National Engineering Research Center for Biomaterials Sichuan University Chengdu 610064 China

**Keywords:** electroactive, hydrogel, myocardial infarction, nanozyme

## Abstract

Injury and apoptosis of cardiomyocytes (CMs) lead to the excessive accumulation of reactive oxygen species (ROS) within the infarcted region, which affects the viability of healthy CMs in the border zone and contributes to the progressive enlargement of the infarct area. The subsequent replacement of necrotic myocardium with fibrotic tissue disrupts normal electrophysiological conduction pathways. This study develops a multifunctional hydrogel, TAlg/PEDOT@PMOF, incorporating an integrated PEDOT@PMOF nanofiller designed to simultaneously scavenge ROS and restore electrical coupling following myocardial infarction (MI). The ROS‐neutralizing capability of the nanofiller stems from the manganese porphyrin, which closely emulates the catalytically active site of native antioxidant enzymes. To increase electrical conductivity, the conductive polymer PEDOT is immobilized onto the MOF structure via a polydopamine (PDA) adhesion layer. Furthermore, the hydrogel network is functionalized with cell‐adhesion peptides, enabling a synergistic enhancement of ROS clearance and electrical signal transmission by the nanofiller at both the cellular and tissue scales. This dual functionality is evidenced by improved cytoprotection under oxidative stress, enhanced calcium transient in cardiomyocytes, restoration of cardiac function, and reduced susceptibility to arrhythmia. These results establish an effective strategy for engineering an integrated enzyme‐mimicking system and highlight a practical and innovative approach for future MI therapy.

## Graphical abstract

An integrated nanoparticle PEDOT@PMOF is constructed as a redox‐active nanozyme and electroactive nanofiller in a cell‐affinity hydrogel platform for acute MI treatment. It is demonstrated that MOF‐based nanozymes have good catalytic ability, and the incorporation of PEDOT increases the conductivity. This work provides a promising strategy to combine ROS‐scavenging and electrical coupling reconstruction therapies for future applications.

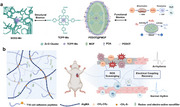

## 1 Introduction

Cardiovascular disease (CVD) continues to be the foremost cause of mortality worldwide, with ischemic heart disease predominantly manifesting as myocardial infarction (MI) affecting more than seven million individuals annually, imposing a substantial burden on global public health systems.^[^ ^1^ ^]^ MI is most commonly caused by stenosis or complete occlusion of the coronary arteries, resulting in inadequate blood perfusion to the myocardial tissue. This lack of perfusion damages the delicate structure of cardiomyocytes, which are highly sensitive to oxygen deprivation, while also causing irreversible impairment to their electromechanical coupling function. Without timely intervention, ischemic necrosis can rapidly extend through the full thickness of the ventricular wall, culminating in MI. Following infarction, the myocardium undergoes a cascade of pathological changes that substantially increase susceptibility to cardiac dysfunction, including angina pectoris and arrhythmias. Over time, negative ventricular remodeling caused by decompensation ultimately progresses to heart failure (HF).^[^ ^2^ ^]^

Reactive oxygen species (ROS) play a central role in the pathophysiological progression of MI. In the acute phase of MI, the cessation of aerobic metabolism leads to widespread cardiomyocyte death, driven by ischemia‐induced necrosis. This is caused by ion pump failure due to reduced ATP hydrolysis, intracellular acidosis, calcium overload, and mitochondrial dysfunction, all of which contribute to excessive ROS production.^[^ ^3^ ^]^ Mitochondria, key regulators of cellular redox homeostasis, are the principal source of ROS in adult cardiomyocytes. Under ischemic injury, upregulation of ROS‐producing enzyme systems combined with impairment of ROS‐detoxifying enzymes generates elevated oxidative stress. Increased ROS levels cause severe injury not only in the ischemic core but also in the border zone between viable and damaged myocardium, accelerating cardiomyocyte death and enlarging the infarcted area. Similar to other ischemic tissue injuries, damaged cardiomyocytes release a variety of endogenous molecules such as cellular debris, nucleic acids, and membrane lipids collectively referred to as damage‐associated molecular patterns (DAMPs). These molecules trigger activation of the immune system, leading to infiltration of the infarcted area by inflammatory cells, including neutrophils and resident cardiac macrophages with pro‐inflammatory phenotypes. Although the initial inflammatory response clears necrotic debris, it also releases a complex array of cytokines and chemokines, both pro‐ and anti‐inflammatory, that further exacerbate myocardial injury.^[^ ^4^ ^]^ During this inflammatory phase, ischemia‐induced ROS production is intimately linked to activation of inflammatory signaling cascades in the infarcted myocardium.^[^ ^5^ ^]^ Furthermore, ROS contributes to post‐MI cardiac remodeling by modulating the expression of matrix metalloproteinases (MMPs), promoting interstitial fibrosis, and inducing cardiomyocyte hypertrophy.^[^ ^6^ ^]^ Therefore, targeted ROS scavenging and modulation of the inflammatory response have emerged as crucial therapeutic strategies in the management of MI.

When ROS production overwhelms endogenous antioxidant defenses in the ischemic myocardium, therapeutic interventions aimed at scavenging ROS become essential. Various pharmacological antioxidants including metformin,^[^ ^7^ ^]^ xanthine oxidase (XO) inhibitors,^[^ ^8^ ^]^ carvedilol,^[^ ^9^ ^]^ and nicorandil^[^ ^10^ ^]^ as well as natural antioxidant enzymes such as catalase (CAT),^[^ ^11^ ^]^ superoxide dismutase 2 (SOD2),^[^ ^12^ ^]^ and glutathione peroxidase (GPx) have shown therapeutic potential. However, pharmacological interventions are often constrained by limitations such as suboptimal bioavailability, dose‐dependent toxicity, short half‐life, and systemic side effects. Natural enzymes, despite their potent activity, face challenges including poor structural stability, environmental sensitivity, and high production costs, which hinder their clinical application. In light of these limitations, nanomaterials that mimic the catalytic structure and function of natural enzymes, often referred to as “nanozymes,” have emerged as a promising alternative for ROS regulation. These include polyphenol‐based nanoparticles, metal nanoparticles, metal oxides, carbon nanomaterials, and metal–organic framework (MOF) materials.^[^ ^13^ ^]^ MOFs, in particular, are attractive due to their well‐defined metal ion coordination centers and organic ligand structures that closely mimic the active sites of natural antioxidant enzymes. This structural similarity enables MOF‐based nanozymes to replicate the catalytic activities of natural enzymes. Moreover, compared to natural enzymes, MOF‐based nanozymes exhibit several advantages, including broad‐spectrum ROS scavenging capability, exceptional stability under physiological and pathological conditions, and highly tunable physicochemical properties such as particle size, surface charge, and specific surface area.^[^ ^14^ ^]^ These properties have led to the widespread exploration of MOF‐based nanozymes for applications in the diagnosis and treatment of inflammatory and ischemic disorders. Among these, MOF systems constructed using manganese porphyrin ligands are of particular interest due to their high metal–ligand stability, the ability of manganese to cycle between multiple oxidation states, and their versatility in reacting with various reactive oxygen and nitrogen species.^[^ ^15^ ^]^

Beyond the early oxidative and inflammatory phase, the post‐infarction myocardium undergoes significant structural remodeling, ultimately forming non‐contractile scar tissue rich in collagen fibers. This fibrotic scar lacks the intrinsic electrical conductivity of native myocardium, leading to disruption of electrical signal propagation across the infarcted region. This discontinuity in electrical coupling between cardiomyocytes results in impaired electromechanical synchronization, regional diastolic dysfunction, and an increased risk of malignant arrhythmias. The importance of electrical conductivity in cardiac tissue engineering has long been recognized, prompting the development of various conductive biomaterials such as conductive polymers, carbon nanotubes, and metallic nanostructures that can be incorporated into hydrogel scaffolds designed to mimic the native extracellular matrix.^[^ ^16^ ^]^ These engineered materials not only improve the biocompatibility and cellular affinity of the scaffold but also increase electrical conduction in infarcted myocardium. However, most MOF materials exhibit inherently poor conductivity due to the insulating nature of their organic ligands. Recent advances have demonstrated that integrating MOFs with conductive polymers can significantly increase electron transport between the internal surface of MOF and external conductive pathways, improving the overall electrical performance of the composite.^[^ ^17^ ^]^

**Scheme 1:**
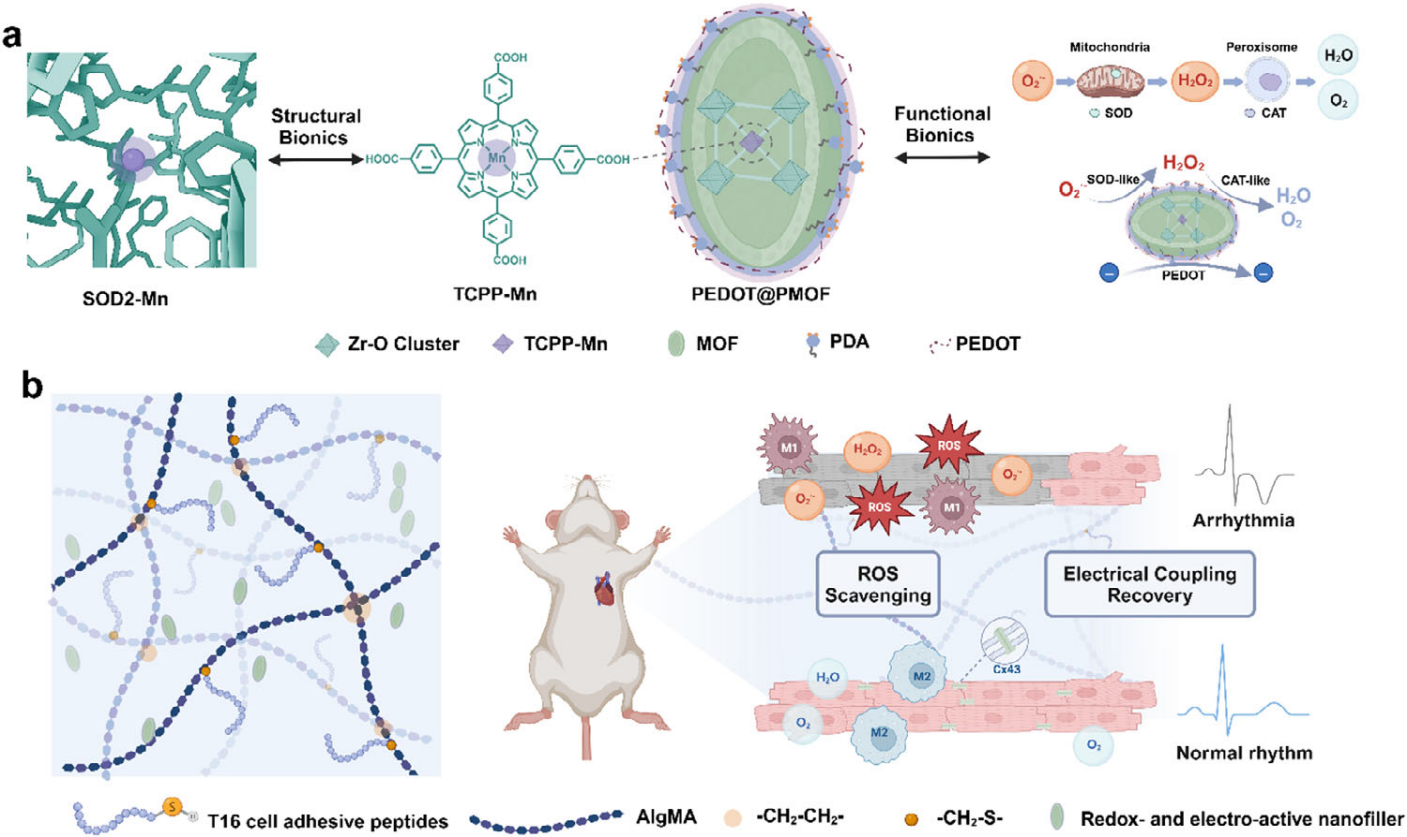
Schematic illustration of a) design strategy and functional components of MOF‐based nanozyme and b) application of the redox‐active and electroactive hydrogel for myocardial infarction treatment by ROS scavenging and electrical coupling recovery.

The present study aimed to address both the oxidative and electrical deficiencies of infarcted myocardium by designing a multifunctional therapeutic platform. Further, a manganese porphyrin‐based MOF nanozyme core that mimics the catalytic architecture of natural SOD2 was synthesized. To improve electrical conductivity and increase biocompatibility, the MOF nanozyme was coated with a polydopamine (PDA) adhesion layer and subsequently conjugated with poly(3,4‐ethylene dioxythiophene) (PEDOT). This composite nanofiller, endowed with both antioxidant and electroconductive properties, was incorporated into a cell‐affinity hydrogel platform to produce a redox‐active and electroactive hydrogel system (**Scheme** **1**). In vitro, the PEDOT@PMOF nanofiller demonstrated both SOD‐like and CAT‐like catalytic activities, enabling efficient ROS scavenging and modulation of macrophage polarization toward an anti‐inflammatory phenotype. The PDA coating conferred favorable biocompatibility and aqueous dispersibility to the MOF core. At the same time, the PEDOT polymer served as a charge‐transfer medium, aligning the conductivity of the hydrogel with that of native myocardium. In vivo, using a rat MI model established by permanent ligation of the left anterior descending coronary artery (LAD), administration of this multifunctional hydrogel significantly reduced ROS levels. Moreover, it improved the inflammatory microenvironment at the infarct site. This intervention effectively prevented expansion of the infarcted region, promoted restoration of cardiac function, and decreased susceptibility to arrhythmias. These findings highlight the therapeutic potential of integrating ROS scavenging with electrical coupling restoration, offering a promising approach for advanced myocardial repair following MI.

## 2 Results and Discussion

### 2.1 Design Strategies and Preparation of TAlg/PEDOT@PMOF Hydrogel

**Figure 1:**
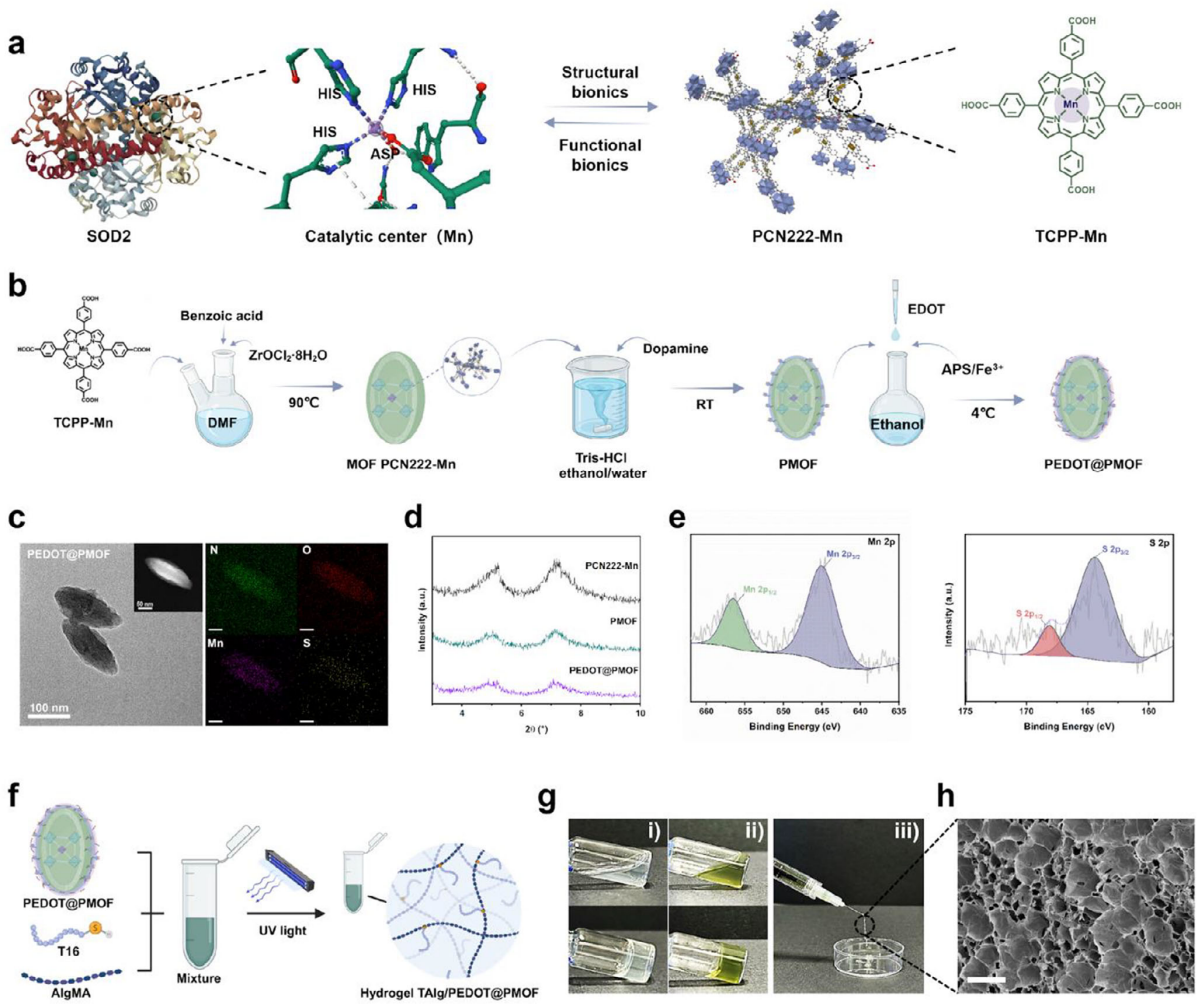
Design strategies and preparation of PEDOT@PMOF nanofiller and TAlg/PEDOT@PMOF hydrogel. a) TCPP‐Mn possessed a bioinspired structure mimicking the catalytic center of the native SOD2. b) Schematic of the synthetic procedure of PEDOT@PMOF. c) TEM and HAADF images of PEDOT@PMOF and element mappings (N, O, Mn, S). Scale bars, 100, 50, and 50 nm. d) PXRD spectra of the MOF PCN222‐Mn, PMOF, and PEDOT@PMOF. e) Mn 2p and S 2p XPS spectra of PEDOT@PMOF. f) Schematics of the formation of the TAlg/PEDOT@PMOF hydrogel. g) The sol–gel transition and injectability of the TAlg/PEDOT@PMOF hydrogel. h) SEM image of TAlg/PEDOT@PMOF. Scale bar, 200 µm.

Redox‐active and electroactive nanofillers (PEDOT@PMOF) were fabricated through a polydopamine‐assisted in situ assembly approach (Scheme S2, Supporting Information). The biomimetic organometallic ligand, manganese(III) tetrakis(4‐carboxyphenyl)porphyrin (TCPP‐Mn), contains an Mn(III) metal center precisely anchored within an N_4_‐macroheterocyclic porphyrin ring (**Figure** **1**a). This coordination environment closely emulates the active site geometry of native antioxidant enzymes such as superoxide dismutase 2 and catalase, suggesting that manganese porphyrin ligands could exhibit enzyme‐like catalytic activity under their structural similarity to biological catalytic domains.^[^ ^13b^ ^]^ The successful synthesis of TCPP‐Mn was confirmed by ^1^H NMR spectroscopy (Figure S1 Supporting Information), where the aromatic ring protons exhibited a chemical shift at 7.28 ppm, and the protons from nitrogen‐bound penta‐heterocycles were observed at 8.86 ppm, both in agreement with reported literature values.^[^ ^13d^ ^]^

The catalytic efficiency of manganese porphyrin is governed by the interplay between the Mn(III) center and the electron distribution within the porphyrin macrocycle. In TCPP‐Mn, the presence of four peripheral deprotonated benzoyloxy groups acts as a strong electron donor, increasing electron density at the Mn(III) center and enhancing redox activity. To further optimize catalytic performance, TCPP‐Mn was utilized as a ligand to coordinate with zirconium–oxo clusters via its peripheral carboxylate groups, forming a highly porous MOF structure. The positively charged zirconium oxygen clusters (Zr–oxo) nodes electrostatically neutralized the negative charges of the TCPP‐Mn ligands, improving charge distribution around the metal center and boosting catalytic reactivity. The resulting nanoscale MOF, PCN222‐Mn, was synthesized and structurally verified by powder X‐ray diffraction (PXRD). As shown in Figure 1d, the PXRD pattern matched well with previously reported PCN222‐Mn profiles, confirming phase purity.^[^ ^13d^ ^]^ Compared with the UV–vis absorption spectrum of free TCPP‐Mn, the MOF exhibited a slight red shift in absorption peaks, likely attributable to enhanced π–π conjugation arising from periodic assembly within the crystalline framework (Figure S2, Supporting Information). Transmission electron microscopy (TEM) imaging (Figure S3, Supporting Information) revealed that the MOF crystals adopted a uniform spindle‐shaped morphology, maintaining excellent structural integrity, with dimensions averaging ≈160 nm along the major axis and 80 nm along the minor axis.

To introduce electroactive properties, poly(3,4‐ethylenedioxythiophene) (PEDOT) was polymerized in situ onto the MOF surface, producing the desired conductive hybrid nanoparticles. Direct PEDOT deposition was inefficient on pristine MOF surfaces due to the absence of reactive functional groups. To address this, MOF particles were pre‐treated in an alkaline dopamine solution, allowing spontaneous self‐polymerization of dopamine to form a uniform PDA nanocoating. This intermediate layer not only improved interfacial adhesion but also imparted a negative surface charge, as reflected in the increased absolute zeta potential values of the PDA‐modified MOF (PMOF) particles (Figure S4, Supporting Information). Consistent with previous studies,^[^ ^17a^ ^]^ the stoichiometric ratio of PEDOT, PDA, and MOF is a critical determinant of the final conductivity of the composite.

PEDOT was synthesized by oxidative polymerization of EDOT monomers in ethanol using Fe^3^⁺ as a catalyst. The initially neutral, undoped PEDOT was subsequently oxidatively doped, forming a conductive polycationic species. Electrostatic interactions and π–π stacking between PEDOT and the PDA‐rich PMOF surface facilitated stable anchoring, producing the integrated electroactive nanofiller PEDOT@PMOF. The morphology of PEDOT@PMOF is presented in Figure 1c. Dynamic light scattering (DLS) analysis indicated hydrodynamic diameters of ≈140 nm for pristine MOF, 180 nm for PMOF, and 220 nm for PEDOT@PMOF, consistent with the incremental increase expected from surface modifications. These values were slightly larger than the corresponding TEM measurements due to hydration layer effects (Figure S5, Supporting Information). Elemental composition and spatial distribution, determined via scanning transmission electron microscopy–energy dispersive spectroscopy (STEM–EDS) mapping, confirmed the uniform distribution of Mn and S elements, validating the incorporation of Mn within the porphyrin core and successful PEDOT polymerization on the MOF surface (Figure 1c). X‐ray photoelectron spectroscopy analysis (Figure 1e; Figure S6, Supporting Information) further corroborated these structural assignments, with characteristic Mn 2p and S 2p signals confirming both the redox‐active and electroactive nature of the composite.

Alginate‐based hydrogels are widely employed in biomedical engineering due to their excellent biocompatibility, modifiable chemical structure, and mild gelation conditions. Conventional ionic cross‐linking with divalent cations such as Ca^2+^, while straightforward, often yields inhomogeneous networks due to rapid, uncontrolled ionic interactions, resulting in poor mechanical consistency.^[^ ^18^ ^]^ To overcome this, alginate can be functionalized with methacrylic anhydride (AlgMA), allowing covalent network formation via light‐induced radical polymerization in the presence of a photoinitiator. This approach not only improves network uniformity but also permits the incorporation of bioactive moieties, such as cell adhesion peptides, to enhance the cellular affinity of otherwise bioinert alginate scaffolds.^[^ ^19^ ^]^ In the present study, AlgMA was synthesized by grafting methacrylate groups onto the alginate backbone, as confirmed by ^1^H NMR (Figure S7, Supporting Information). To further promote cardiomyocyte adhesion, the cysteine‐terminated T16 peptide (Cys–T16) was prepared through standard solid‐phase peptide synthesis. Blending of AlgMA with Cys–T16, followed by photoinitiated cross‐linking under UV irradiation, resulted in the formation of a three‐dimensional peptide‐functionalized hydrogel network (Figure 1f). The complete synthetic pathway and chemical characterization of the hydrogel are detailed in Scheme S3, Supporting Information.

Upon encapsulation of the PEDOT@PMOF nanofillers, the resulting composite hydrogel, TAlg/PEDOT@PMOF, exhibited both redox activity and electroactivity. As shown in Figure 1g, the hydrogel demonstrated reversible sol–gel transition behavior and excellent injectability, making it well‐suited for minimally invasive cardiac delivery. Scanning electron microscopy (SEM) imaging (Figure 1h) revealed an interconnected 3D porous network, providing a structurally robust yet compliant scaffold. This architecture offers sufficient mechanical integrity while enabling uniform distribution of embedded nanofillers, creating a favorable microenvironment for myocardial repair applications. The cell adhesion and biological performance of the hydrogel will be comprehensively evaluated in Section 2.4.

### 2.2 In Vitro SOD‐ and CAT‐Like Activity of TAlg/PEDOT@PMOF Hydrogel

**Figure 2:**
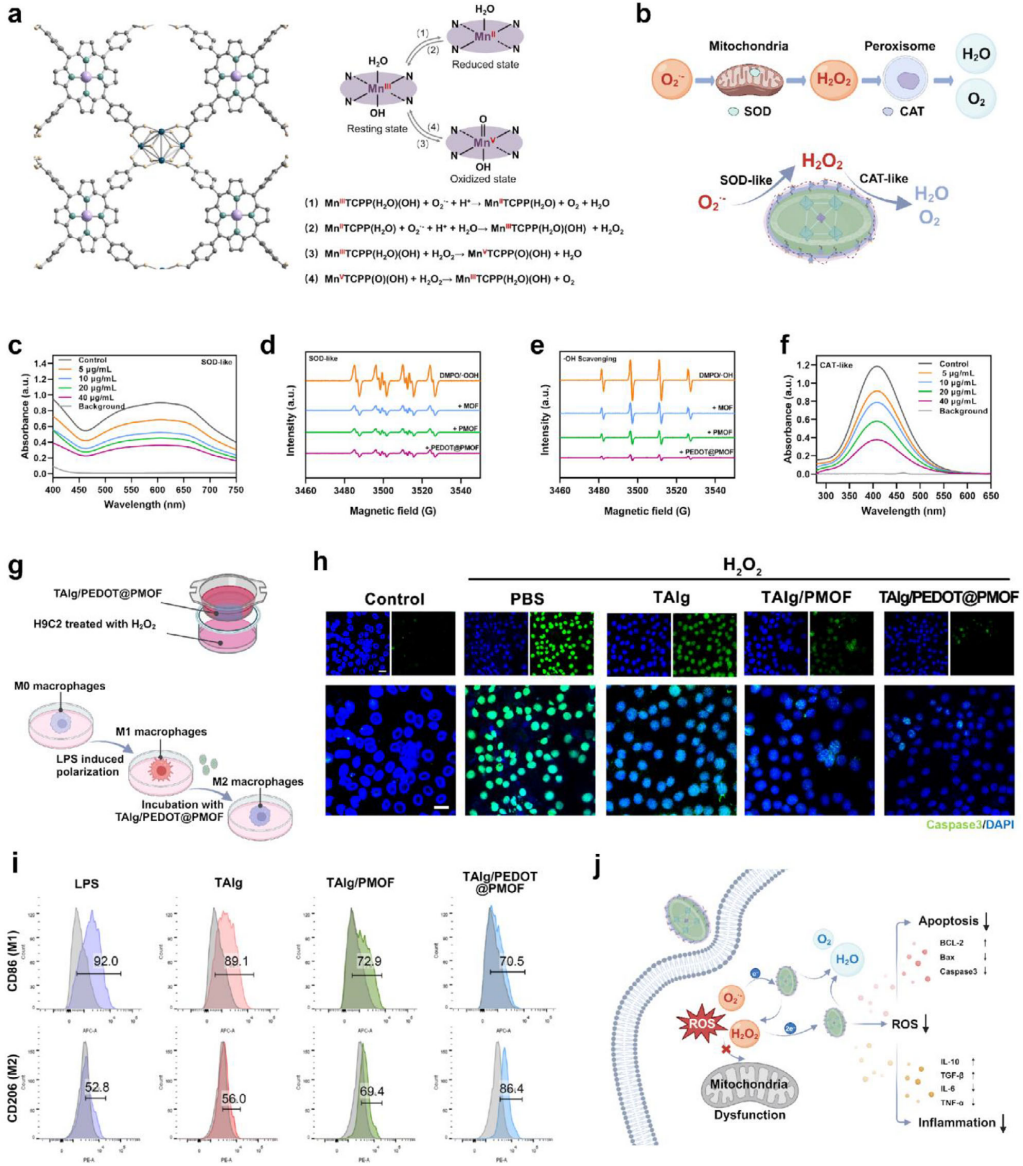
In vitro enzyme‐like catalytic efficiency and catalytic mechanism of redox‐active nanofiller PEDOT@PMOF and cellular antioxidative efficiency of TAlg/PEDOT@PMOF hydrogel. a) Scheme for the interconversion among resting, reduced, and oxidized states of TCPP‐Mn, as well as the detailed chemical reactions in each step of conversion. (Purple ball: Mn, dark green ball: Zr, green ball: N, yellow ball: O, gray ball: C). b) Schematics of multiple free radical production in vivo. c) UV–vis absorption spectra of O_2_^·−^ reflecting the SOD‐like activity of PEDOT@PMOF at different concentrations. d) EPR for ·OOH elimination situation of MOF, PMOF, and PEDOT@PMOF at 6 min after examination. e) EPR for ·OH elimination situation of MOF, PMOF, and PEDOT@PMOF at 6 min after examination. f) UV–vis absorption spectra of H_2_O_2_ reflecting the CAT‐like activity of PEDOT@PMOF at different concentrations. g) Schematic representation of the experimental treatment of H9C2 and RAW264.7 cells. h) Confocal microscopy images of Caspase3 (green) in H9C2 cells. Scale bar, 40 µm. i) Flow cytometry data of acticated RAW264.7 by LPS with different treatments and quantification of M1 (CD86) and M2 (CD206). j) Schematic representation of the mechanism by which the ROS scavenging effect of the PEDOT@PMOF nanofillers reduced apoptosis and suppressed inflammation.

The coordination environment of Mn(III) in TCPP‐Mn closely resembles the active domains of metal cofactor‐dependent enzymes such as SOD2 and CAT (Figure 1a). Guided by structure–activity principles, supported by extensive previous studies^[^ ^20^ ^]^ and validated by these experiments, the manganese porphyrin metal–organic linker demonstrates biomimetic SOD‐ and CAT‐like activities (**Figure** **2**a). By organizing TCPP‐Mn as a catalytic unit and using highly positively charged zirconium oxide clusters as coordination nodes, the electron cloud density around Mn(III) is reduced, yielding PCN222‐Mn, a MOF core with redox activity. Following myocardial ischemia, the balance between intracellular ROS production and clearance is disrupted. Excessive accumulation of superoxide and hydrogen peroxide inflicts severe damage on cells and tissues adjacent to the ischemic region, expanding the infarct area. To evaluate the ROS scavenging capacity of PEDOT@PMOF in vitro, its activity against multiple ROS types was examined (Figure 2b). In mitochondrial respiration, electron leakage from the electron transport chain leads to the formation of superoxide.^[^ ^21^ ^]^ The SOD‐like activity of the nanofillers was assessed using a xanthine (X)–xanthine oxidase (XO) system, in which generated superoxide radicals reduce the chromogen NBT to blue formazan with a strong absorbance at 560 nm. As shown in Figure 2c, increasing PEDOT@PMOF concentrations corresponded with a progressive decrease in absorbance intensity, indicating dose‐dependent superoxide scavenging. EPR measurements further confirmed this effect. In methanol, DMPO trapped superoxide radicals, producing a characteristic six‐peak spectrum with four large and two small peaks in the absence of nanofillers. Upon addition of MOF, PMOF, or PEDOT@PMOF, peak intensity diminished significantly, and in the PEDOT@PMOF group, the superoxide signal was significantly weakened over time (Figure 2d; Figure S8, Supporting Information).

Superoxide anions can further react with hydrogen ions to form hydroxyl radicals. In vitro, hydroxyl radicals generated via the Fenton reaction react with salicylic acid to form a chromogenic complex. As shown in Figure S9 (Supporting Information), increasing concentrations of PEDOT@PMOF progressively reduced the maximum absorbance at 510 nm. EPR spectra displayed four peaks characteristic of hydroxyl radicals (1:2:2:1 ratio) after 6 min of microwave irradiation (Figure 2e). Addition of MOF, PMOF, or PEDOT@PMOF sequentially weakened these signals, with complete disappearance of the radical peaks in the PEDOT@PMOF group within 12 min (Figure S10, Supporting Information). Under pathological conditions, the conversion of oxygen to water is impaired, and superoxide anions are converted to hydrogen peroxide by SOD2. The CAT‐like activity of the nanomaterials was evaluated using the redox reaction of titanium sulfate with hydrogen peroxide, producing a peroxide–titanium complex detectable at 405 nm. As shown in Figure 2f, absorbance decreased progressively with increasing PEDOT@PMOF concentration, confirming its efficient hydrogen peroxide scavenging activity.

Based on previous literature, the mechanism underlying the redox activity of PEDOT@PMOF was investigated. Similar to natural SOD2,^[^ ^22^ ^]^ the catalytic activity of metalloporphyrins arises from their extended π‐conjugation and the redox transitions of the central metal atoms. In the resting state, the Mn(III) center of manganese porphyrin is coordinated by four nitrogen atoms and two solvent molecules (H_2_O/OH^−^) in a trans‐axial arrangement (Figure 2a). Disproportionation of O_2_·^−^ involves Mn(III)/Mn(II) cycling, forming the Mn(II)TCPP(H_2_O) transition state, which subsequently releases oxygen and produces water. The catalytic disproportionation of H_2_O_2_ proceeds via Mn(III)/Mn(V) transitions. Initially, H_2_O_2_ undergoes deprotonation to form a perhydroxide anion (HOO^−^), which replaces an axial hydroxyl ligand in Mn(III)TCPP(H_2_O)(OH^−^), generating the Mn(V) oxidation state. Mn(V) then returns to its resting Mn(III) form, with H_2_O_2_ dismutated to H_2_O and O_2_.

In the present experiments, modification of the MOF structure with conductive PEDOT further improved catalytic activity. This observation is consistent with a previous study indicating that MOF surface modifications can significantly alter catalytic performance.^[^ ^23^ ^]^ For example, noble metal nanoparticles incorporated on or within MOFs can create additional active sites, improving catalytic efficiency, whereas thick polymer coatings may risk pore blockage and compromise structural integrity.^[^ ^24^ ^]^ In this study, conductive polymer modification was selected to exploit potential synergy between the electronically conjugated PEDOT chains and the MOF framework. Literature^[^ ^17a^ ^]^ combined with the current experimental findings suggests that the improved activity of PEDOT@PMOF arises from two complementary redox processes operating within the nanoconfined structure. First, the MOF core undergoes Mn(III)/Mn(II)/Mn(V) transitions intrinsic to the manganese porphyrin catalytic cycle. Second, PEDOT chains function as charge‐transfer bridges, facilitating interfacial Faradaic processes^[^ ^17^ ^]^ and amplifying the overall redox activity. This dual mechanism contributes to the improved enzyme‐mimetic catalytic performance of PEDOT@PMOF.

### 2.3 Cellular Anti‐Apoptosis and Inflammatory Phenotypes Regulation Ability of TAlg/PEDOT@PMOF Hydrogel

The antioxidant and anti‐apoptotic properties of the TAlg/PEDOT@PMOF hydrogel were evaluated using an in vitro oxidative stress model, in which rat cardiomyocytes (H9C2) were exposed to H_2_O_2_. The optimal nanofiller concentration was first determined. Cell viability assays showed that increasing nanofiller content improved H9C2 survival under oxidative stress; however, excessively high concentrations reduced cell viability under normal culture conditions (Figure S11, Supporting Information). Based on these findings and literature reports,^[^ ^13d^ ^]^ 20 µg mL^−1^ was selected for subsequent experiments. Oxidative injury was induced by incubating cells with 200 µM H_2_O_2_ for 1 h, followed by treatment with hydrogel extracts (Figure 2g). Caspase‐3, a key effector protease in apoptosis,^[^ ^25^ ^]^ was examined via immunofluorescence staining. H_2_O_2_ exposure triggered substantial apoptosis in H9C2 cells, and hydrogels lacking nanofillers provided negligible protection. In comparison, TAlg/PEDOT@PMOF significantly reduced apoptosis (Figure 2h). Flow cytometry confirmed these results: late apoptosis decreased from 23.7% in the PBS group to 6.13% in the TAlg/PEDOT@PMOF group (Figure S12, Supporting Information). Western blot analysis further demonstrated that TAlg/PEDOT@PMOF treatment upregulated the anti‐apoptotic protein BCL‐2 while downregulating NF‐κB, Bax, and Caspase‐3 expression (Figure S13, Supporting Information).

Progression of post‐ischemic MI involves multiple biochemical pathways, with inflammation playing a central role. Elevated ROS levels drive macrophage polarization toward the M1 pro‐inflammatory phenotype, leading to the secretion of cytokines such as IL‐6, IL‐1β, and TNF‐α, which impair tissue recovery. To investigate the immunomodulatory effect of the hydrogel, an LPS‐induced RAW264.7 macrophage polarization model was established. Cells were treated with hydrogel extracts and labeled with APC‐CD86 (M1 marker) and PE‐CD206 (M2 marker). Flow cytometry revealed a reduction in the CD86⁺/Blank ratio from 92.0% in the LPS group to 70.5% in the TAlg/PEDOT@PMOF group, indicating reduced M1 polarization. The CD206⁺/Blank ratio increased from 52.8% to 86.4%, suggesting promotion of the M2 anti‐inflammatory phenotype (Figure 2i). Cytokine analysis further supported these findings. In the TAlg/PEDOT@PMOF group, IL‐6 and TNF‐α levels were significantly lower, while IL‐10 and TGF‐β levels were elevated compared to PBS controls (Figure S14, Supporting Information). These results demonstrate that TAlg/PEDOT@PMOF hydrogel not only mitigates oxidative stress–induced apoptosis but also modulates inflammatory responses by suppressing pro‐inflammatory signaling and increasing anti‐inflammatory cytokine production.

### 2.4 Electroactive Hydrogel Promotes Cell‐Matrix Affinity and Intracellular Calcium Transient

The physical properties and biochemical signaling motifs of a hydrogel matrix determine its interaction with cells and tissues.^[^ ^26^ ^]^ Achieving intimate contact between the hydrogel and the target tissue is essential for functional integration. To assess structural stability, the rheological properties of the hydrogels were examined. In oscillatory mode, the shear modulus of the TAlg/PEDOT@PMOF hydrogel was ≈1700 Pa (Figure S15, Supporting Information), sufficient to provide mechanical support to the ventricular wall. The network structure disrupted by large shear forces rapidly recovered, indicating the self‐healing capacity of the hydrogel.

**Figure 3:**
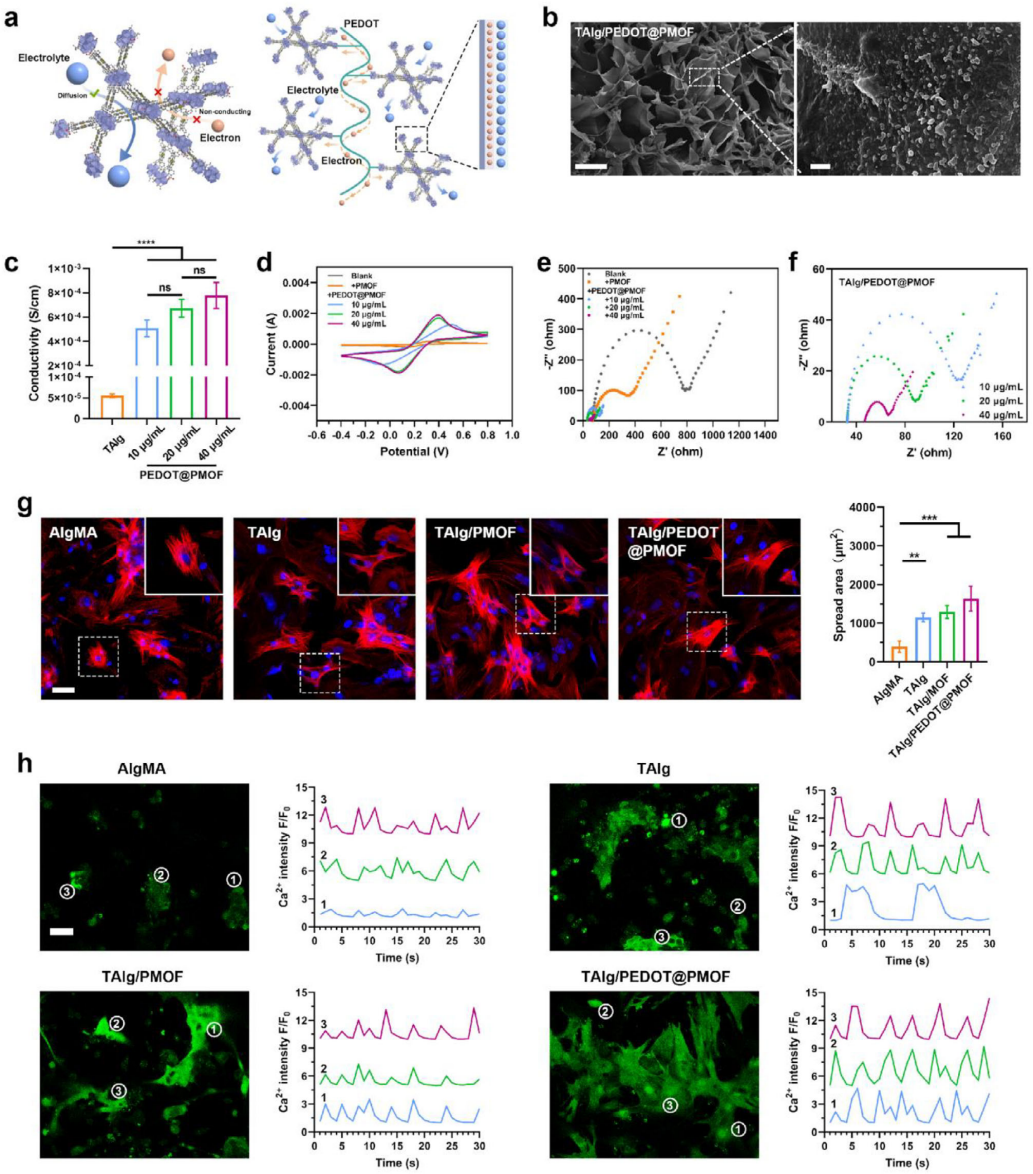
Electrical activity of the TAlg/PEDOT@PMOF hydrogel and the promotion of maturation of NRCMs in 2D culture. a) Schematic of the mechanism by which the conductive polymer PEDOT modification improved the conductivity of the MOF. In pure MOF crystals, only electrolytes can diffuse in and out of the MOF pores, while electrons cannot migrate along the framework. However, after incorporating the conductive polymer PEDOT with the MOF, both electrons and electrolytes gain access to the MOF surface, forming an electrochemical double‐layer structure at the MOF interface. b) SEM image of the cross‐section of the lyophilized TAlg/PEDOT@PMOF hydrogel and the nanofillers on its skeleton. Scale bars, 200 and 2 µm. c) Conductivity of the different hydrogels. d) CV curve of different hydrogels. e,f) Nyquist curve of different hydrogels. g) Confocal images of the cytoskeletons of NRCMs on different hydrogels. Scale bar, 40 µm. h) Ca^2+^ transient of NRCMs on different hydrogels. Representative images from confocal microscopy and plots of intracellular Ca^2+^ transient changes with time. Scale bar, 40 µm. Data are presented as mean ± s.d., and One‐way ANOVA with Tukey's multiple comparisons test was used to compare the means of the values of groups. ns indicates not significant, *p < 0.05, **p < 0.01, *** *p* < 0.001, and****p < 0.0001.

Electrical conductivity was introduced by incorporating electroactive PEDOT@PMOF nanofillers. As the MOF core has limited intrinsic conductivity, PEDOT was polymerized in situ following surface modification with an adhesive PDA layer. The conjugated PEDOT chains acted as charge transfer bridges, releasing electrons into the electrolyte during electrochemical processes and enhancing the Faradaic response at the interface, therefore contributing pseudocapacitance.^[^ ^17^ ^]^ Uniform dispersion of conductive nanofillers (e.g., metal nanoparticles, carbon nanotubes, graphene, MXene) within the hydrogel matrix is known to form interconnected electron pathways, improving conductivity (**Figure** **3**a).^[^ ^25^, ^27^ ^]^ In the TAlg/PEDOT@PMOF hydrogel, π–π stacking and C–H···π interactions facilitated the formation of conductive networks. Resistivity was measured by the four‐probe method for hydrogels with fixed volume and cross‐sectional area. The conductivity of TAlg/PEDOT@PMOF was calculated as 7.78 ± 0.06 × 10^−4^ S cm^−1^. As shown in Figure 3c, incorporation of PEDOT@PMOF significantly increased conductivity, reaching values comparable to native myocardial tissue.^[^ ^28^ ^]^ Electrochemical measurements further supported these findings. Cyclic voltammetry (CV) indicated that PEDOT‐modified nanofillers greatly improved electrochemical performance, with the TAlg/PEDOT@PMOF hydrogel showing a larger CV curve area compared to TAlg and TAlg/PMOF, attributable to the synergistic pseudocapacitance of PEDOT, PDA, and MOF (Figure 3d). Electrochemical impedance spectroscopy (EIS) demonstrated a smaller semicircle diameter in the Nyquist plot for TAlg/PEDOT@PMOF, indicating reduced charge‐transfer resistance (Figure 3e,f).

Previous studies have shown that hydrogel matrices with improved bioactivity more effectively mimic the native cellular microenvironment, supporting cell adhesion and integration with host tissue.^[^ ^29^ ^]^ Functional nanofillers further enable efficient chemical and electrical signal transmission between cells and surrounding tissue, amplifying therapeutic effects. To evaluate the potential of the TAlg/PEDOT@PMOF hydrogel grafted with the cell‐adhesive T16 peptide for tissue repair, its impact on neonatal rat cardiomyocyte (NRCM) growth was investigated. Cytoskeletal staining of NRCMs cultured in 2D on the hydrogel surface was performed to assess adhesion morphology and cell spreading using confocal microscopy. As shown in Figure 3g, NRCMs on the unmodified AlgMA hydrogel showed poor adhesion, small spreading areas, and significant cell aggregation. In comparison, the T16 peptide–grafted hydrogel provided abundant adhesion sites, promoting well‐organized cytoskeletal structures. The T16 peptide sequence (CGERGAPGFRGPAGPNGIPGEKGPAGERGAP) is derived from repetitive motifs of recombinant human collagen type III (rhCol III).^[^ ^30^ ^]^ A similar previous study confirmed the bioactivity of rhCol III–derived peptides and partially elucidated their adhesion mechanism.^[^ ^31^ ^]^ Within T16, motifs such as GER (glycine–glutamic acid–arginine) and GEK (glycine–glutamic acid–lysine), where oppositely charged residues are adjacent, are considered critical for high cell adhesion activity. A previous study has shown that T16‐mediated adhesion is competitively inhibited by the RGD peptide,^[^ ^30^ ^]^ suggesting a similar integrin‐binding mechanism. However, the specific binding sites and structural modes require further investigation.

Stable electrical coupling between cardiomyocytes is essential for coordinated cardiac contractility, with rhythmic contraction–relaxation cycles in NRCMs driven by intracellular Ca^2+^ fluctuations. To assess the impact of the TAlg/PEDOT@PMOF hydrogel matrix on Ca^2+^ conduction and excitation–contraction coupling, real‐time Ca^2+^ transients were qualitatively analyzed using the fluorescent indicator Fluo‐4 (Figure 3h; Videos S1–S4, Supporting Information). Cardiomyocytes isolated from neonatal rat left ventricles were seeded onto hydrogel scaffolds. As shown in Figure 3h, Ca^2+^ fluorescence traces from three randomly selected regions revealed that NRCMs cultured on AlgMA and TAlg hydrogels displayed asynchronous beating. In comparison, those on TAlg/PEDOT@PMOF surfaces showed synchronous activity with evident spontaneous electrical signals. Peak amplitude and pattern analysis further showed that the TAlg/PEDOT@PMOF group maintained rhythmic Ca^2+^ transients with higher signal intensity, in comparison to the irregular, lower‐intensity signals observed in the AlgMA and TAlg groups. Intervals between Ca^2+^ peaks indicated that NRCMs in the TAlg/PEDOT@PMOF group had shorter Ca^2+^ release and reuptake times (Figure S16, Supporting Information). Overall, the electroactive TAlg/PEDOT@PMOF hydrogel improved intercellular electrical coupling, likely by bridging resistive barriers and facilitating ionic communication between adjacent cells, supporting synchronized and rhythmic cardiomyocyte contractions.

### 2.5 In Vivo ROS Scavenging Ability of the TAlg/PEDOT@PMOF Hydrogel

**Figure 4:**
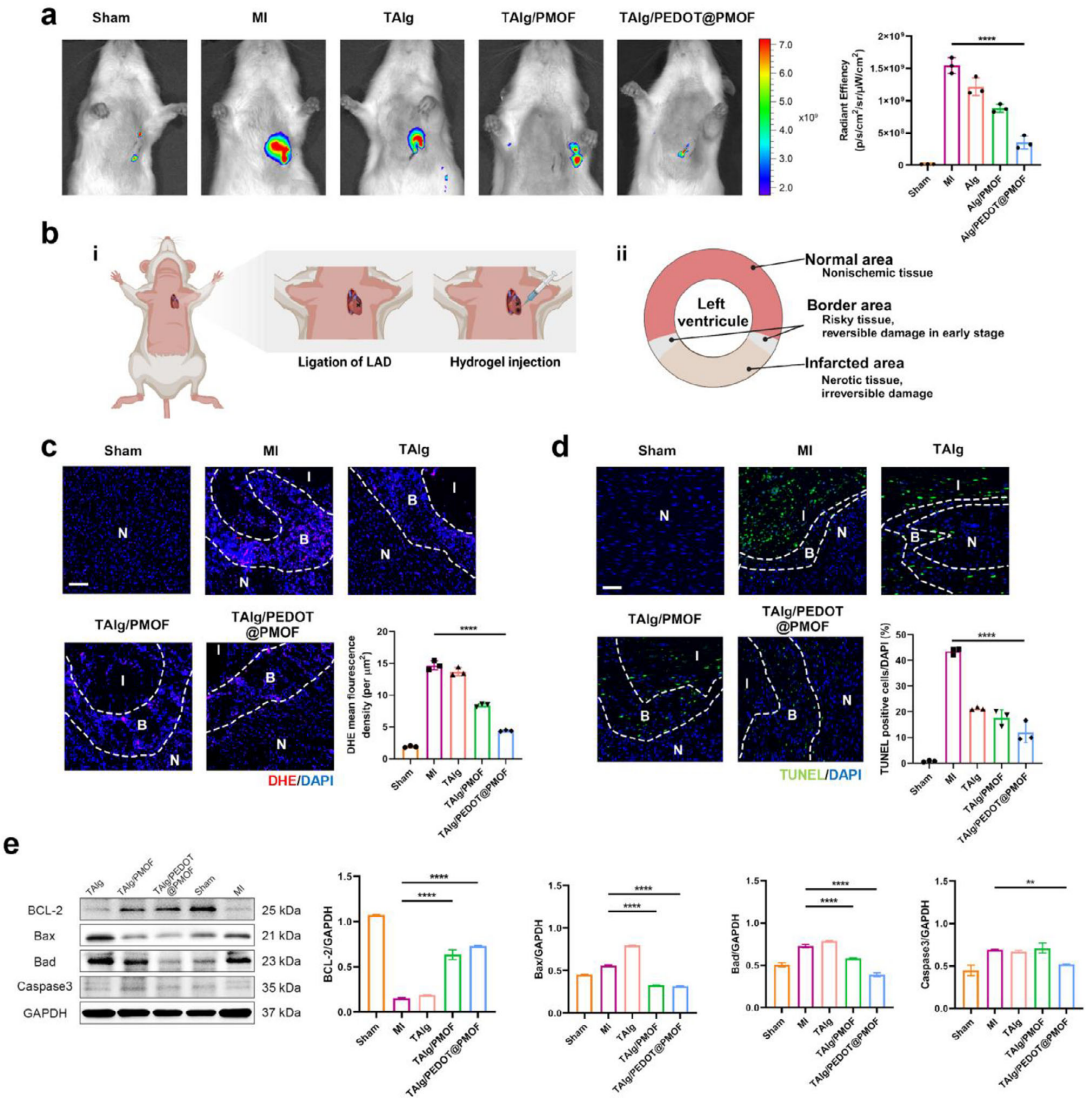
In vivo ROS scavenging ability of the TAlg/PEDOT@PMOF hydrogel. a) In vivo imaging of H_2_O_2_ and quantitative analysis of fluorescence intensity. b) The establishment of animal models for MI and injection of TAlg/PEDOT@PMOF hydrogels, and a schematic representation of the left ventricular cross‐section after MI. c) DHE staining of heart sections 3 days after injection. “N” indicates normal area, “B” indicates border area, and “I” indicates infarcted area. d) TUNEL staining of heart sections 3 days after injection. e) The protein expression of BCL‐2, Bax, Bad, and Caspase3 in myocardial tissues treated with different hydrogels. Data are presented as mean ± s.d., and One‐way ANOVA with Tukey's multiple comparisons test was used to compare the means of the values of groups. **p < 0.01 and ****p < 0.0001.

Cell‐based assays confirmed the ROS‐scavenging capacity of the TAlg/PEDOT@PMOF hydrogel, prompting evaluation of its therapeutic efficacy in vivo at the site of MI. Among intracellular ROS, O_2_·^−^ is a primary reactive species, generated by enzymes such as NADH oxidase, lipoxygenase, and xanthine oxidase in cardiomyocytes. Under physiological conditions, endogenous antioxidant defenses, including GPx4, maintain redox homeostasis. However, during early ischemia, the abrupt ROS surge overwhelms these systems, leading to lipid peroxidation, protein inactivation, and DNA damage. Given the demonstrated SOD‐ and CAT‐like activities of PEDOT@PMOF in vitro, this study examined its ability to scavenge ROS in infarcted myocardium. In vivo imaging using the H_2_O_2_‐sensitive near‐infrared probe Ampliflu Red showed an increase in H_2_O_2_ fluorescence in the MI group compared to the Sham group (**Figure** **4**a). Treatment with TAlg/PEDOT@PMOF hydrogel substantially attenuated this signal. A moderate reduction in H_2_O_2_ fluorescence was also observed in the TAlg and TAlg/PMOF groups, indicating that bioactive hydrogel scaffolds or MOF‐loaded hydrogels alone confer partial cardioprotection through ROS clearance. ROS levels in myocardial cryosections were further assessed using the superoxide‐specific fluorescent probe DHE. The MI group showed intense red fluorescence in the infarct region, consistent with elevated superoxide levels. In comparison, fluorescence intensity was significantly lower in the TAlg/PEDOT@PMOF group (Figure 4c), confirming its strong ROS‐scavenging effect in vivo.

Apoptotic cell death in myocardial tissue was evaluated by immunofluorescence staining (Figure 4d) and Western blot analysis. Similar to the in vitro oxidative injury model, MI hearts showed increased expression of pro‐apoptotic proteins Bax, Bad, and Caspase‐3. Following TAlg/PEDOT@PMOF hydrogel injection, the anti‐apoptotic protein BCL‐2 was upregulated (Figure 4e), indicating that ROS neutralization by the hydrogel mitigates apoptosis in ischemic myocardium. Excess ROS also promotes cytokine and chemokine expression, driving recruitment of inflammatory cells into the infarct zone, where they exacerbate tissue injury.^[^ ^32^ ^]^ To assess macrophage polarization in vivo, CD206 (M2 marker) and iNOS (M1 marker) immunostaining was performed. TAlg/PEDOT@PMOF treatment significantly increased M2 macrophage infiltration while limiting M1 macrophage prevalence (Figure S17, Supporting Information). Furthermore, cytokine profiling of myocardial homogenates revealed elevated pro‐inflammatory IL‐6 and TNF‐α in MI tissue. In comparison, the TAlg/PEDOT@PMOF group showed significantly higher IL‐10 expression (Figure S18, Supporting Information), consistent with a shift toward an anti‐inflammatory milieu.

### 2.6 Improvement in Cardiac Function and Morphology after MI

**Figure 5:**
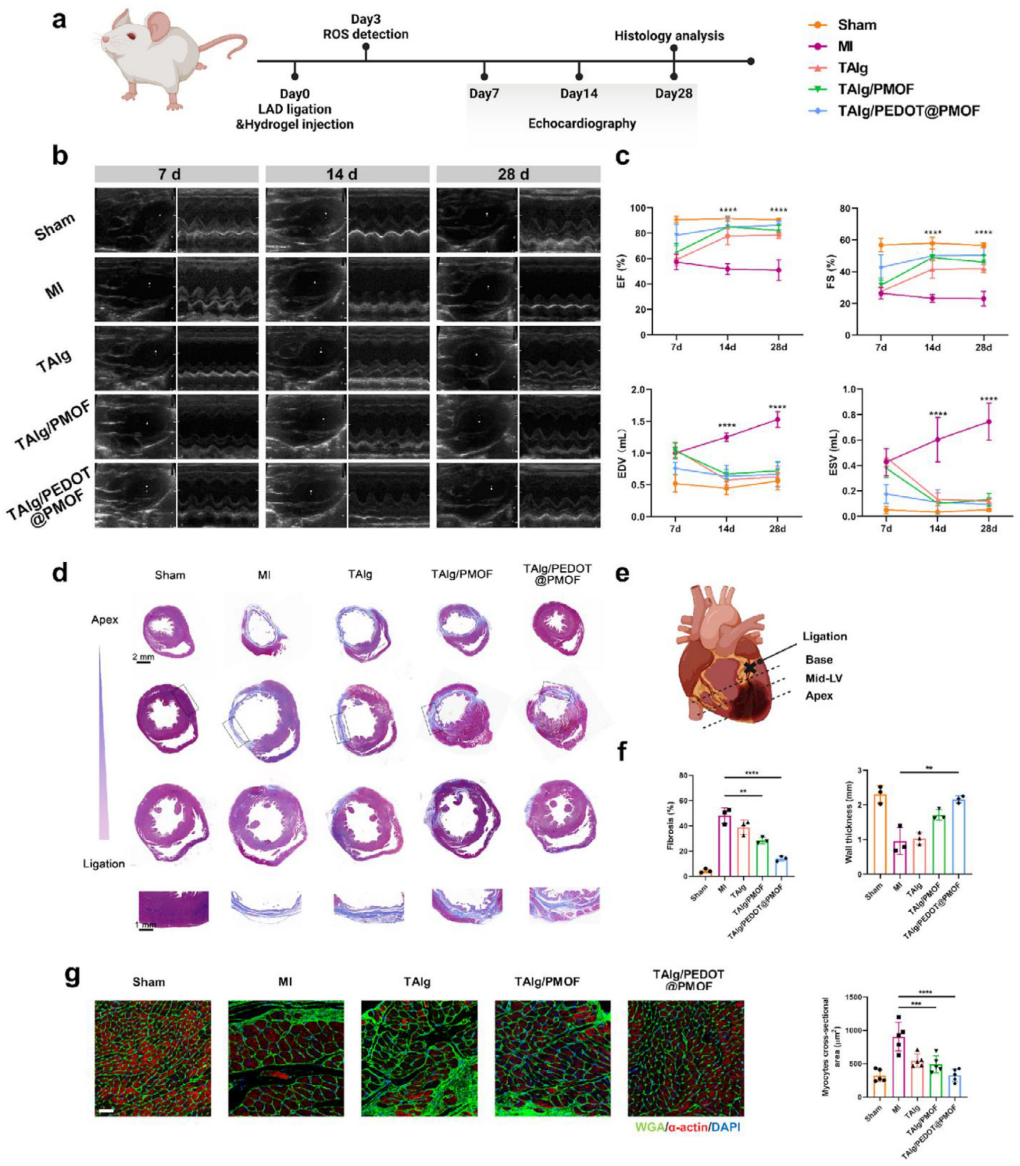
Therapeutic effects of TAlg/PEDOT@PMOF hydrogel in the MI rat model. a) Timeline of the MI model establishment, hydrogel injection treatment, and cardiac function evaluation. b) Echocardiographic images of the rats in different treatment groups. c) Cardiac functional parameters assessment, including EF, FS, EDV, ESV (n = 6). d) Masson's trichrome staining images of infarcted hearts in different groups at 28th days. Scale bars, 2 and 1 mm. e) Schematic of cardiac pathological sections from the apex to the base of the heart. f) Quantitative analysis of fibrosis degree and wall thickness of infarcted LV (n = 3). g) WGA and α‐actin staining of the cross‐section of cardiomyocytes in different groups and quantitative analysis. Scale bar, 50 µm. Data are presented as mean ± s.d., and One‐way ANOVA with Tukey's multiple comparisons test was used to compare the means of the values of groups. **p < 0.01, *** *p* < 0.001, and ****p < 0.0001.

Following acute MI, the initial oxidative burst and inflammatory response rapidly give way to irreversible cardiomyocyte loss within the ischemic zone. As collagen fibers accumulate, non‐contractile scar tissue progressively replaces viable myocardium, severely impairing cardiac performance and predisposing to HF.^[^ ^33^ ^]^ Various experiments were performed to evaluate the long‐term therapeutic impact of TAlg/PEDOT@PMOF hydrogel on cardiac function. Echocardiography was conducted at 7, 14, and 28 days post‐MI induction and hydrogel treatment (**Figure** **5**a,b). By day 7, all MI‐operated groups revealed thinning of the left ventricular wall relative to Sham, accompanied by reduced ejection fraction (EF) and fractional shortening (FS), reflecting impaired ventricular structure and contractile function. TAlg/PEDOT@PMOF treatment partially restored EF and FS values, with end‐diastolic volume (EDV) indicating better preservation of ventricular geometry without significant dilation. At day 28, 2D echocardiograms revealed pronounced thinning of the anterolateral wall in the MI group compared with Sham. In comparison, the TAlg/PEDOT@PMOF group showed attenuated wall thinning. Functional parameters at this time point confirmed sustained benefit: EF and FS had improved significantly by day 14 and stabilized at favorable levels by day 28. These findings suggested that TAlg/PEDOT@PMOF hydrogel supports early myocardial repair, preserves chamber size, and mitigates pathological remodeling.

Cardiac morphology and histopathology were further examined on day 28. Masson's trichrome staining of three serial sections (from apex to papillary muscle) demonstrated full‐thickness infarction of the left ventricular wall in MI hearts, with dense collagen deposition throughout the infarct region, increased ventricular volume, and evidence of pathological remodeling compared to Sham (Figure 5d). In the TAlg/PEDOT@PMOF group, wall thickness was better maintained, fibrosis was reduced, and remodeling was minimal. Wheat germ agglutinin (WGA) staining of cardiomyocyte membranes at the infarct border zone revealed increased hypertrophy in the MI group, as the non‐infarcted myocardium compensated for the loss of contractile tissue. In comparison, cardiomyocyte cross‐sectional area was significantly smaller in the TAlg/PEDOT@PMOF group (Figure 5g). A similar antihypertrophic effect was also observed in TAlg and TAlg/PMOF groups, likely attributable to the mechanical support conferred by the hydrogel scaffold, which helped maintain uniform stress distribution and reduced compensatory hypertrophy.^[^ ^34^ ^]^

### 2.7 Regulation of Cardiac Electrophysiology after MI

**Figure 6:**
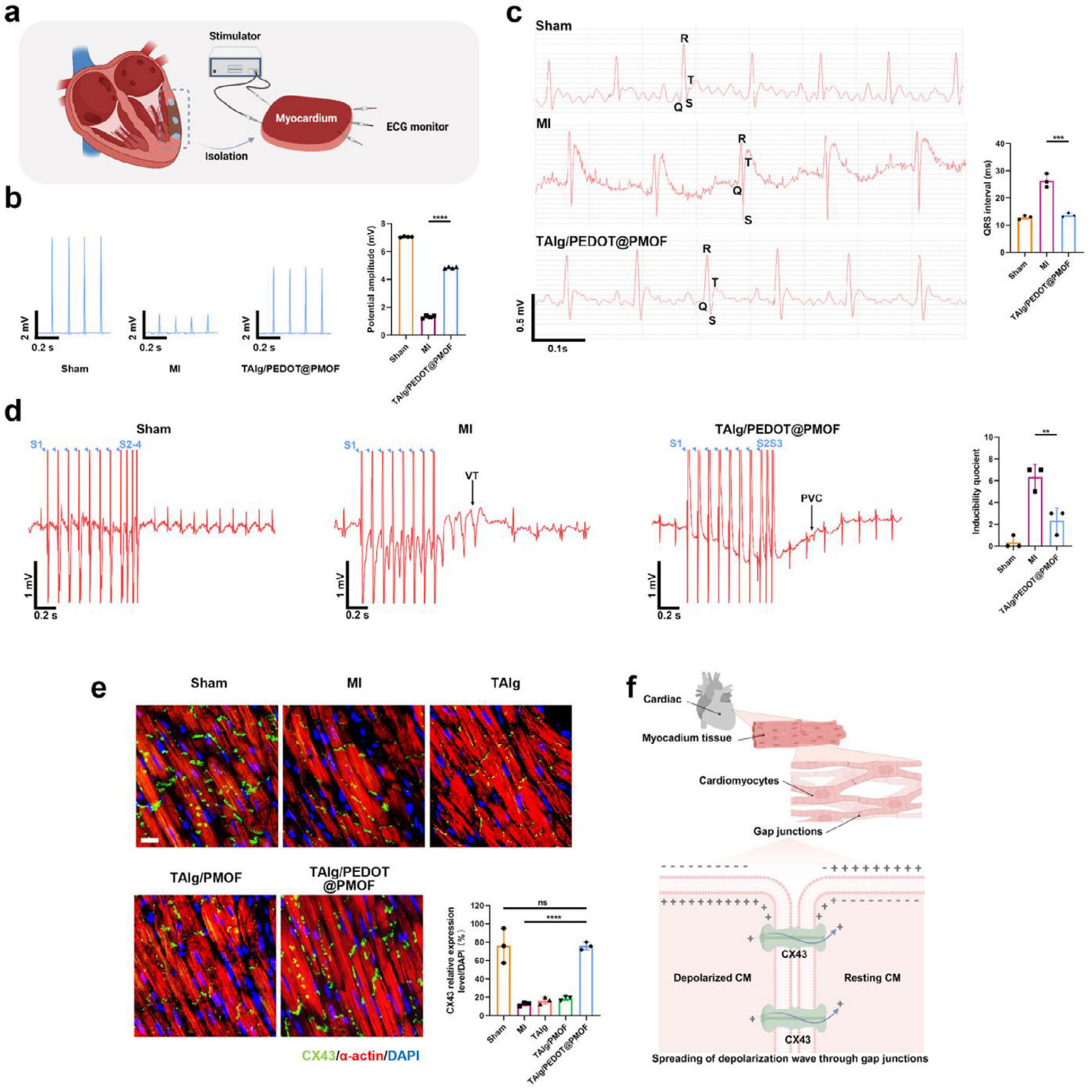
The regulation capacity of TAlg/PEDOT@PMOF hydrogel of cardiac electrophysiology after MI. a) Schematic diagram of electrical pulse conduction experiments in isolated myocardial tissue. b) Electrocardiograms from different groups and quantitative average local field potential amplitudes from different groups (n = 4). c) Representative ECGs from different groups 28 days after injection and the effect of different hydrogels on QRS duration (n = 3). d) ECGs of arrhythmias induced by PES 28 days after injection and calculated inducibility quotients from different groups (n = 3). e) CX43 and α‐actin staining in different groups. Scale bar, 40 µm. Quantitative analysis reflects the CX43 protein expression in each group (n = 3). Data are presented as mean ± s.d., and One‐way ANOVA with Tukey's multiple comparisons test was used to compare the means of the values of groups, **p < 0.01, ***p < 0.001, and ****p < 0.0001.

The cyclic contraction and relaxation of the heart, essential for maintaining systemic circulation, rely on the excitation–contraction coupling of cardiomyocytes. In vitro findings demonstrated that the TAlg/PEDOT@PMOF hydrogel shows electrical conductivity comparable to native myocardium, supports the maturation of NRCMs on its surface, and facilitates intercellular ion exchange, highlighting its potential in myocardial tissue repair. At four weeks post‐treatment, isolated myocardial tissues were subjected to electrical pulse stimulation, and an electrocardiograph recorder was used to evaluate the effects of different hydrogel implants on pulse propagation (**Figure** **6**a). The TAlg/PEDOT@PMOF group displayed significantly higher local potential amplitude than the MI group, indicating effective restoration of myocardial electrical connectivity in treated hearts (Figure 6b). The hydrogel promoted signal conduction between viable cardiomyocytes within the infarct zone and those in adjacent border areas.

The QRS complex duration in ECG is influenced by heart size, cardiomyocyte architecture, Purkinje network structure, and depolarizing ionic currents.^[^ ^35^ ^]^ After MI, delayed ventricular depolarization and the insulating nature of scar tissue prolong the QRS interval. Analysis revealed that QRS duration was significantly shorter in the TAlg/PEDOT@PMOF group compared with the MI group (Figure 6c), with statistically significant intergroup differences. Ventricular arrhythmias and sudden cardiac death are major post‐MI complications, particularly in the early months after infarction. Electrical heterogeneity between surviving myocytes and fibrotic tissue in the border zone disrupts depolarization and repolarization, predisposing to arrhythmias. To evaluate arrhythmia susceptibility, programmed electrical stimulation (PES) was applied in vivo. As shown in Figure 6d, Sham animals maintained a normal ECG waveform after three additional stimuli. In comparison, MI rats developed non‐sustained ventricular tachycardia following bursts of stimulation, whereas TAlg/PEDOT@PMOF‐treated rats only showed isolated non‐sustained ventricular premature contractions after two extra stimuli. These results indicate that TAlg/PEDOT@PMOF implantation significantly lowers arrhythmia susceptibility under PES.

This improved electrical integration likely involves multiple mechanisms. First, post‐MI inflammatory microenvironments exacerbate conduction heterogeneity in the border zone; the redox‐active TAlg/PEDOT@PMOF hydrogel mitigates this by scavenging ROS during early treatment. Second, the electroactive hydrogel provides a conductive bridge across non‐conductive scar tissue, restoring electrical coupling between isolated viable myocytes and healthy myocardium, thus reducing depolarization re‐entry.

Loss of viable cardiomyocytes disrupts action potential propagation and predisposes to arrhythmia. Gap junction proteins, particularly connexin‐43 (CX43), are critical for intercellular electrical communication (Figure 6f). CX43 expression and function are influenced by intracellular pH, Ca^2+^ concentration, phosphorylation status, transmembrane voltage, and neurohumoral factors.^[^ ^36^ ^]^ In the MI group, CX43 expression in the infarct border zone was significantly reduced, impairing electrophysiological coordination. In comparison, TAlg/PEDOT@PMOF treatment preserved CX43 expression (Figure 6e), promoting synchronized electrical activity. To track nanofiller distribution after intramyocardial injection, PEDOT@PMOF was labeled with indocyanine green (ICG), a near‐infrared fluorescent dye. Fluorescence imaging showed gradual signal attenuation over three days, with almost complete clearance by day 7 (Figure S19, Supporting Information), indicating biodegradation and clearance from myocardial tissue. Acute toxicity assessment of PEDOT@PMOF in vivo showed no evidence of organ damage; H&E staining of major organs collected 48 h post‐injection revealed no pathological changes compared with controls (Figure S20, Supporting Information).

## 3 Conclusion

In summary, the present study successfully developed a core–shell nanocomposite, PEDOT@PMOF, as a multifunctional redox‐active nanozyme and electroactive nanofiller incorporated into a cell‐affinity hydrogel platform for acute MI therapy. This therapeutic strategy is designed to simultaneously address two central pathological processes following MI: excessive free radical accumulation in the acute phase and impaired electrical coupling in the chronic remodeling stage. The catalytic functionality of the nanozyme is derived from the precisely ordered manganese porphyrin (TCPP‐Mn) ligands within the MOF lattice, which structurally and functionally emulate the catalytic domain of endogenous SOD2. While unmodified MOFs typically suffer from low electrical conductivity due to the limited delocalization between organic linkers and metal clusters, this constraint was overcome by in situ polymerization of the conductive polymer PEDOT onto the MOF surface, facilitated by an intermediate PDA adhesion layer. Integration of this tailored nanofiller into a T16 peptide–modified hydrogel further increased biointerface compatibility, optimizing cell–matrix interactions to achieve improved functional performance in biological environments.

Extensive in vitro characterization under various oxidative stress conditions confirmed that PEDOT@PMOF exhibits potent catalytic activity and effective ROS scavenging. This redox performance is mediated by dynamic Mn(II/III/V) valence transitions within the porphyrin framework and is further amplified by efficient Faradaic charge transfer between the MOF core and PEDOT chains. The presence of the T16 peptide within the hydrogel scaffold promoted neonatal rat cardiomyocyte adhesion, spreading, cytoskeletal organization, and robust calcium transients, all indicative of improved cardiomyocyte maturation and electromechanical coupling. In vivo, administration of the TAlg/PEDOT@PMOF hydrogel in a rat MI model resulted in significant therapeutic benefit: during the early post‐MI phase, its ROS‐scavenging capacity mitigated oxidative injury and preserved viable cardiomyocytes within the peri‐infarct zone; in the subsequent reparative phase, its electroconductive properties facilitated reestablishment of intercellular electrical communication, markedly reducing susceptibility to arrhythmia.

This study represents an innovative advancement in the integration of SOD‐mimetic MOF nanozymes with conductive polymers, achieving improved catalytic and electroactive performance through synergistic material design. The resulting multifunctional hydrogel exerts a dual therapeutic effect: 1) biochemical modulation by ameliorating oxidative stress and the inflammatory microenvironment, and 2) electrophysiological restoration by providing a conductive pathway for synchronized electrical signal transmission. By aligning the structural and functional properties of the biomaterial with the physiological and pathological demands of the myocardium, this approach offers a transformative platform for myocardial repair. From the acute reduction of oxidative stress to the progressive reestablishment of stable electrical conduction, this synergistic strategy holds substantial promise as a next‐generation therapeutic platform for cardiac tissue regeneration. However, as with all conductive hydrogel systems currently at the preclinical stage, rigorous long‐term investigations are warranted to evaluate their durability, sustained efficacy, and safety profile in vivo. Moreover, the development of strategies to mitigate potential conduction disturbances arising from the implantation of electroactive biomaterials will be essential to ensure safe and effective clinical translation.

## 4 Experimental Section

Detailed methods are provided in the Supporting Information.

## Conflict of Interest

The authors declare no conflict of interest.

## Supporting information



Supporting Information

Supplemental Video 1

Supplemental Video 2

Supplemental Video 3

Supplemental Video 4

## Data Availability

The data that support the findings of this study are available from the corresponding author upon reasonable request.
