# Supplementary material for: A Redox‐Active and Electroactive Hydrogel Enabled by an Integrated PEDOT@PMOF Nanofiller for Post‐Infarct Myocardial Repair
Source: Adv Sci (Weinh). 2025 Sep 12;12(43):e05612. doi: 10.1002/advs.202505612 (PMC12631919; doi:10.1002/advs.202505612)
Supplement: Supplementary file 1 — Supporting Information [file ADVS-12-e05612-s005.docx]

Supporting Information

A Redox-Active and Electroactive Hydrogel Enabled by an Integrated PEDOT@PMOF Nanofiller for Post-Infarct Myocardial Repair

Shuyi He, Linyu Long, Wenqi Liu, Zhicun Wang, Fengxiao Wu, Li Yang*, Yunbing Wang

Materials, cells, and animals

Methyl p-formylbenzoate, pyrrole, MnCl_2_·4H_2_O, and ZrOCl_2_·8H_2_O were purchased from Aladdin Reagent Co., Ltd. (Shanghai, China). Lithium phenyl(2,4,6-trimethylbenzoyl) phosphinate (LAP) and ammonium persulfate (APS) were obtained from Sigma-Aldrich Chemical Reagent Co., Ltd. Sodium alginate was sourced from Yuanye Bio-Technology Co., Ltd. (Shanghai, China). At the same time, methacrylic anhydride (MA) was supplied by Macklin Biochemical Technology Co., Ltd. (Shanghai, China). Fetal bovine serum (FBS) and penicillin–streptomycin were purchased from Gibco (NY, USA). Lipopolysaccharide (LPS) and nitro blue tetrazolium (NBT) were obtained from Yeasen Biotechnologies (Shanghai, China). H9C2 and RAW264.7 cell lines were provided by Yuchun Biology (Shanghai, China). Sprague Dawley (SD) rats and neonatal rats were supplied by Dossy Experimental Animal Co., Ltd. (Chengdu, China).

**Characterization**

Proton nuclear magnetic resonance (^1^H NMR) spectra were acquired using a Bruker AMX-400 spectrometer operating at 400 MHz. Fourier transform infrared (FT-IR) spectra were recorded on a Thermo Fisher Nicolet iS10 spectrometer. Ultraviolet–visible (UV–Vis) spectra were measured with a Shimadzu UV-2401PC spectrophotometer. X-ray photoelectron spectroscopy (XPS) analyses were performed using a Thermo Scientific K-Alpha system. Powder X-ray diffraction (PXRD) patterns were obtained on a Bruker D8 Advance diffractometer. Particle size distribution and zeta potential of nanoparticles were determined by dynamic light scattering (DLS) using a Malvern Zetasizer Nano ZS. The morphology of nanoparticles was observed with a Hitachi H-600 transmission electron microscope (TEM) operated at 100 kV, while hydrogel morphology was examined using a Thermo Apreo 2C scanning electron microscope (SEM).

**Experimental Section**

*Synthesis of TCPP-Mn*

TCPP-Mn was synthesized following a previously reported method.^[1]^ First, the organic ligand TPP-COOMe was prepared. Methyl p-formylbenzoate (14 g) was dissolved in 200 mL of propionic acid, and pyrrole (6 mL) was added dropwise under stirring. The mixture was refluxed in the dark for 12 h. Upon completion, the reaction was cooled to room temperature, and the product was collected by filtration, washed three times with methanol and tetrahydrofuran, and vacuum-dried to yield a purple solid.

Next, the metalloporphyrin ligand TCPP-Mn was synthesized. TPP-COOMe (0.854 g) and MnCl_2_·4H_2_O (2.5 g) were dissolved in 100 mL of dimethylformamide (DMF) and refluxed in the dark for 6 h. After cooling to room temperature, 150 mL of water was added, and the mixture was stirred for 1 h. The resulting precipitate was collected by filtration, washed with water three times, and dried. The obtained solid (0.75 g) was then dissolved in a 1:1 mixture of tetrahydrofuran (THF, 25 mL) and methanol (MeOH, 25 mL). An aqueous solution of KOH (2.63 g, 46.95 mmol, 25 mL) was added, and the reaction mixture was refluxed for 12 h. After cooling to room temperature, THF and MeOH were removed by rotary evaporation. Water was added to the residue and heated until complete dissolution. The solution was then acidified with 1 M HCl, inducing gradual precipitation. Acid addition continued until no further precipitate formed. The solid product was collected by filtration, washed thoroughly with water, and vacuum-dried to afford TCPP-Mn as a purple powder.

*Synthesis of MOF material PCN222-Mn*

The nanoscale MOF compound PCN222-Mn was synthesized according to the reported method.^[2]^ The ZrOCl_2_·8H_2_O (30 mg), benzoic acid (0.28 g), and Mn-TCPP (10.98 mg) were dissolved in 14 mL of DMF and heated at 90 °C under stirring for 5 h. Upon completion, the reaction mixture was cooled to room temperature, and the resulting particles were collected by centrifugation. The solids were washed three times with DMF and deionized water, then dispersed in ethanol for storage.

*Synthesis of PEDOT@PMOF*

First, the surface of the MOF material was functionalized with polydopamine (PDA). Dopamine (100 mg) was dissolved in 100 mL of Tris–HCl buffer (pH 8.5) and allowed to pre-polymerize for 30 min. Subsequently, MOF (200 mg) was added to the pre-polymerization solution and polymerized for 12 h. The product was washed three times with ethanol to yield PDA-modified PCN-222-Mn, referred to as PMOF. Next, the conductive polymer PEDOT was coated onto the PMOF surface using a modified method based on a previously reported procedure.^[3]^ Disperse the prepared PMOF (296.4 mg) and EDOT (228 μL) in ethanol (30 mL) under vigorous stirring. Subsequently, add dropwise a solution of FeCl_3_·6H_2_O (6g) and APS (1.5g) in ethanol (10 mL) and stir for 24 hours in an ice bath until the assembly of EDOT on the surface of PMOF is completed. After washing with deionized water and centrifuging, the final nanoparticles, PEDOT@PMOF, were obtained.

*Synthesis of TAlg and preparation of TAlg/PEDOT@PMOF hydrogel*

Firstly, methacrylated sodium alginate (AlgMA) was prepared according to the previously reported procedure.^[4]^ A 1% (w/v) sodium alginate solution was prepared by dissolving 1 g of sodium alginate in 100 mL of deionized water. While maintaining the temperature at 4 °C, 8 mL of methacrylic anhydride was added dropwise, and the pH was kept between 8 and 9 using 5 M NaOH. The reaction was allowed to proceed for 24 h, after which the mixture was dialyzed against water for three days and subsequently freeze-dried to yield AlgMA. The T16 peptide, with the amino acid sequence CGERGAPGFRGPAGPNGIPGEKGPAGERGAP, was synthesized using standard solid-phase peptide synthesis techniques.

The optimal nanofiller concentration in the hydrogel was determined using the CCK8 cell viability assay. Hydrogels containing nanofiller at concentrations of 5, 10, 20, and 40 μg mL^-1^ were prepared. After 48 h of extraction following the established protocol, the extracts were co-cultured with both untreated cells and cells pre-exposed to 200 μM H_2_O_2_ for another 48 h. Comparative analysis showed that the hydrogel containing 20 μg mL^-1^ nanofiller provided the most pronounced cytoprotective effect under oxidative stress, while maintaining a cell viability above 80%. Therefore, this concentration was selected for subsequent experiments. The T16 peptide-to-AlgMA ratio was determined based on previous studies.^[5]^

The TAlg/PEDOT@PMOF hydrogel was prepared by homogeneously dispersing AlgMA (50 mg), the bioactive peptide T16 (5 mg), the photoinitiator LAP (5 mg), and PEDOT@PMOF nanofiller (20 μg) in 1 mL of water, followed by UV-induced crosslinking under 405 nm irradiation for 30–60 seconds. Control hydrogels (TAlg and TAlg/PMOF) were fabricated using the same procedure, with TAlg prepared without nanofiller and TAlg/PMOF containing only PMOF as the nanofiller.

*Evaluation of enzyme-like activity*

The SOD-like activity of PEDOT@PMOF was evaluated using a xanthine/xanthine oxidase (X/XO) system. Nanofillers (20 μg mL^-1^) were combined with X (0.15 mM) and XO (0.02 U mL^-1^) in Tris-HCl buffer (pH 7.0). After 5 min co-incubation at room temperature, 0.05 mM nitro blue tetrazolium chloride (NBT) was added, and absorbance was recorded at 550 nm. Superoxide anion scavenging was further confirmed by electron paramagnetic resonance (EPR) spectroscopy using 5,5-dimethyl-1-pyrroline-N-oxide (DMPO) as the spin-trapping agent, with superoxide generated from the X/XO system. Reaction systems containing MOF, PMOF, or PEDOT@PMOF were analyzed at 2, 6, and 12 min.

Catalase-like (CAT-like) activity was assessed in an H_2_O_2_/Ti(SO_2_)_2_ system. Nanofillers (20 μg mL⁻¹) were mixed with 10 mM H₂O₂ in 2 mL PBS. Aliquots (50 μL) of the mixture were combined with 100 μL Ti(SO_2_)_2_ solution (prepared by dissolving 319.2 mg Ti(SO_2_)_2_ in 8.33 mL H_2_SO_4_ and 51.33 mL ultrapure water). After 5 min incubation, absorbance at 405 nm was measured to determine residual H_2_O_2_ concentration.

Hydroxyl radical (·OH) scavenging activity was evaluated in an H_2_O_2_/FeSO_4_/salicylic acid (SA) system. Hydroxyl radicals were generated via the Fenton reaction between FeSO_4_ (0.1 mmol mL^-1^) and H_2_O_2_ (100 mM). A 40 μL aliquot was added to a mixture containing nanofillers (20 μg mL^-1^) and SA (100 mg mL^-1^) to reach a total volume of 1 mL. The reaction between SA and ·OH produced 2,3-dihydroxybenzoic acid, which was quantified by measuring absorbance at 510 nm after 5 min at room temperature. EPR spectroscopy, using DMPO as the spin-trapping agent, was also performed to confirm ·OH scavenging, with signals monitored at 2, 6, and 12 min.

*Characterization of the rheological behavior of TAlg/PEDOT@PMOF hydrogel*

The rheological behavior of the hydrogels was evaluated using an Anton Paar Modular Compact Rheometer (MCR). Samples of AlgMA, TAlg, and TAlg/PEDOT@PMOF hydrogels (1 mL each) were prepared for analysis. A 25 mm parallel plate geometry was used. In the strain sweep (amplitude sweep) mode, the frequency was fixed at 1 Hz, while the strain was increased from 0.1% to 1000%. The relationship between strain and both the storage modulus (G′) and loss modulus (G″) was recorded. To examine self-healing performance, strain values beyond the flow point (large amplitude) and within the linear viscoelastic range (small amplitude) were alternated to assess recovery behavior.

*Electrical properties of TAlg/PEDOT@PMOF hydrogel*

Following established protocols, the resistivity of thin-layer hydrogel samples was measured using a four-probe resistivity testing system (RTS-8). Conductivity was then calculated using the equation σ = ρ⁻¹, where σ denotes conductivity and ρ denotes resistivity. Cyclic voltammetry (CV) was performed on a CHI760E electrochemical workstation in a three-electrode configuration, with a glassy carbon electrode coated with a thin hydrogel layer as the working electrode, Ag/AgCl as the reference electrode, and platinum as the counter electrode. CV curves were recorded in 0.01 M neutral PBS over a potential range of −0.4 V to 0.8 V at a scan rate of 100 mV s^-1^. Electrochemical impedance spectroscopy (EIS) was carried out at the open-circuit potential in 0.5 M KCl containing 5.0 mM [Fe(CN) _6_]^3-^ to evaluate charge transfer resistance in the hydrogels. The frequency range was set from 10^-2^ Hz to 10^4^ Hz with an amplitude of 5 mV.

*Apoptosis assay on H9C2 cells*

H9C2 cells were seeded in confocal dishes and cultured at 37 °C with 5% CO_2_ until reaching approximately 70% confluence. To induce oxidative stress and mimic *in vivo* conditions, cells were exposed to 200 μM H_2_O_2_ for 1 h. Following stimulation, the medium was replaced with extracts from TAlg, TAlg/PMOF, or TAlg/PEDOT@PMOF hydrogels, and incubation was continued for 24 h. Cells were then stained with Caspase-3/DAPI following the manufacturer's protocol and imaged using a laser confocal microscope. Fluorescent images were recorded for analysis.

In parallel, apoptosis and cell death were assessed by flow cytometry. H9C2 cells were treated as described above, harvested, and stained with PI/Annexin V–Alexa Fluor 488 before flow cytometric analysis.

*Polarization of macrophages*

RAW 264.7 macrophages were stimulated with LPS (100 ng mL^-1^) for 24 h to induce polarization toward the M1 phenotype. In parallel, extracts from the respective hydrogel groups were added to the culture and incubated for another 24 h. Cells were then harvested, stained sequentially with APC–CD86, fixed, permeabilized, and subsequently stained with PE–CD206. Macrophage phenotypes were analyzed by flow cytometry.

*2D culture of primary NRCMs on* *TAlg/PEDOT@PMOF hydrogel*

Primary cardiomyocytes were isolated from 1‑day‑old SD rats to evaluate the interaction between NRCMs and TAlg/PEDOT@PMOF hydrogels modified with cell adhesion peptides. Ventricular tissues were enzymatically digested, and the cardiomyocytes were seeded onto glass‑bottom dishes coated with AlgMA, TAlg, or TAlg/PEDOT@PMOF hydrogels. After the cells adhered, spread, and established stable beating, cytoskeletal staining was performed according to the manufacturer's instructions. Cellular morphology was subsequently examined using a confocal microscope.

*Ca^2+^ transient behavior of NRCMs on* *TAlg/PEDOT@PMOF hydrogel*

The NRCMs were cultured for 7 days on the surfaces of AlgMA, TAlg, and TAlg/PEDOT@PMOF hydrogels. Rhythmic cell beating was observed under a light microscope. Cells were then stained with Fluo‑4 AM to monitor calcium flux, which was visualized, imaged, and recorded using a confocal microscope. Fluorescence intensity (F) was normalized to baseline (F0), and time‑dependent calcium transients were analyzed and plotted using ImageJ software.

*Establishment of the MI model and hydrogel treatment*

All animal procedures were approved by the Medical Ethics Committee of Sichuan University (Approval No. K2023010). Male Sprague–Dawley rats (120 g) were used for MI modeling and *in vivo* therapeutic evaluation. Rats were anesthetized with a small-animal inhalation anesthesia system, the fur on the left thorax was shaved, and the skin was disinfected with iodophor. A transverse incision was made between the 4th and 5th ribs, followed by a small longitudinal thoracotomy. The heart was gently exteriorized using forceps, and the opening was stabilized. A 6‑0 nylon suture was used to perform a non-transmural ligation of the left anterior descending (LAD) coronary artery 1–2 mm below the left auricle. All procedures were performed under sterile conditions, and body temperature was maintained at 37 °C. Continuous ECG monitoring was carried out, with ST–T segment elevation and pallor or dark-gray discoloration of the anterior LV wall confirming successful MI induction. Subsequently, 30 μL of hydrogel was injected into both the center and border zones of the infarction site using a 27G needle. The heart was promptly returned to the thoracic cavity, and the chest was closed and sutured. The wound was disinfected with iodophor. Following recovery, rats were returned to their cages, labeled, and assigned to the Sham, MI, TAlg, TAlg/PMOF, or TAlg/PEDOT@PMOF groups. Sham-operated rats underwent the same thoracotomy and closure procedures without LAD ligation.

*ROS scavenging efficacy in vivo*

The H₂O₂-sensitive near-infrared probe Ampliflu Red (1 mM) was incorporated into hydrogels of each treatment group. Following MI induction, rats received intramyocardial injections of the probe-loaded hydrogels, while the Sham group was injected with PBS containing the same probe concentration. At 24 h post-injection, rats were anesthetized, and near-infrared signals (571–585 nm) were captured using the IVIS Spectrum (PerkinElmer) imaging system. Fluorescence intensity was quantified with Bruker MI SE software.

On day 3 after hydrogel administration, rats were anesthetized, euthanized, and hearts were collected and fixed in 4% paraformaldehyde. Samples were embedded in paraffin and sectioned transversely at 4 µm thickness for TUNEL immunofluorescence staining. Frozen heart sections were used for dihydroethidium (DHE) staining to assess ROS levels. Protein expression levels of BCL‑2 (BA0412), Bax (BA0315‑2), Bad (10435‑1‑AP), Caspase‑3 (19677‑1‑AP), IL‑10 (GB11108), TNF‑α (ab307164), and IL‑6 (ab259341) were determined by immunoblotting, with GAPDH (AB0037) as an internal control.

*Cardiac function parameters monitored by echocardiography*

At 1, 2, and 4 weeks after hydrogel treatment, echocardiography was performed to evaluate left ventricular (LV) function. A Philips CX50 ultrasound system with an L15‑7io high‑frequency probe was used to record cardiac parameters for each group. Rats were anesthetized with isoflurane, placed supine with limbs secured, and chest hair removed. Using 2D-guided M-mode echocardiography, images were obtained from the parasternal long-axis view at the papillary muscle level. Ejection fraction (EF), fractional shortening (FS), LV end‑diastolic volume (EDV), and LV end‑systolic volume (ESV) were measured, with each value averaged over three consecutive cardiac cycles.

*Histological and immunofluorescence analysis*

Seven days after injection treatment, rat hearts were harvested. Paraffin‑embedded heart tissues were sectioned horizontally and stained for iNOS to identify M1 macrophages, while CD206 staining was performed to detect M2 macrophages. At four weeks post‑treatment, hearts were again collected. Paraffin‑embedded hearts were sectioned transversely along planes perpendicular to the line from the apex to the base. Three sections, spanning from the LV apex to the papillary muscle level, were subjected to Masson staining. Left ventricular wall thickness was measured as the average distance between the endocardium and epicardium at both ends and the center of the scar. Cross‑sections at the mid‑papillary muscle level were stained with wheat germ agglutinin (WGA) for cell membrane visualization, connexin 43 (CX43) to detect myocardial gap junction proteins at the infarct–normal tissue border, and cardiac troponin I (cTnI) for myocardial troponin localization. Nuclei were counterstained with DAPI. All images were captured using a fluorescence microscope and analyzed quantitatively with ImageJ software.

*Cardiac electrophysiological assessment*

The electrical conduction capacity of infarcted tissue was evaluated four weeks post‑treatment. Animals were euthanized, and LV scar tissue was excised. Electrical pulses were delivered using an electrical stimulator (YC‑3) at 10 mV and 1 Hz, transmitted through the infarcted myocardium *in vitro*, and recorded by an electrocardiographic monitor. QRS interval duration was also assessed. Four weeks after treatment, an electrocardiography (ECG) was performed to measure the QRS complex duration. Rats were anesthetized, allowed to stabilize for five minutes, and ECG waveforms were recorded using three surface electrodes (negative, positive, and ground).

Arrhythmia susceptibility was examined via programmed electrical stimulation (PES). PES studies were performed with an isolated programmable stimulator, and arrhythmia induction was scored according to a standardized induction quotient. The clinical PES protocol included pacing at the spontaneous rhythm (cycle length 120 ms) followed by single (70 ms), double (60 ms), and triple (50 ms) extra stimuli. The induction quotient was scored as follows:

0: No premature ventricular contractions (PVCs) or ventricular tachycardia (VT)

1: Non‑sustained PVCs or VT (≤15 beats) induced by triple extra stimuli

2: Sustained PVCs or VT (>15 beats) induced by triple extra stimuli

3: Non‑sustained PVCs or VT induced by double extra stimuli

4: Sustained PVCs or VT induced by double extra stimuli

5: Non‑sustained PVCs or VT induced by a single extra stimulus

6: Sustained PVCs or VT induced by a single extra stimulus

7: Sustained or non‑sustained PVCs or VT induced by eight stimulation sequences

8: Asystole after pacing termination

Higher scores reflected greater arrhythmia susceptibility.

*Clearance and biosafety of PEDOT@PMOF in vivo*

To track nanofillers within the hydrogel after implantation, indocyanine green (ICG), a near‑infrared fluorescent dye, was used to label the nanofillers. Specifically, 10 mg of PEDOT@PMOF and 2 mL of ICG solution were added to 20 mL of distilled water and stirred overnight at room temperature. After centrifugation, the supernatant was removed, and the precipitate was washed with distilled water to obtain ICG‑labeled PEDOT@PMOF, which was then incorporated into the hydrogel.

Following hydrogel injection in rats, *in vivo* fluorescence imaging was performed at multiple time points using a multimodal small animal imaging system (*In‑Vivo* FX PRO, Bruker, Germany). The fluorescence intensity and dynamic distribution of PEDOT@PMOF nanofillers were analyzed with Bruker MI SE software. For biosafety evaluation, an aqueous PEDOT@PMOF suspension was directly injected into the myocardium, and major organs were subsequently analyzed histologically. At 48 hours post‑injection, rats were euthanized, and the heart, liver, spleen, lungs, and kidneys were collected for H&E staining.

**Statistical Analysis**

All quantitative results are expressed as mean ± standard deviation from independent experiments. The sample size (n) for each analysis is indicated in the corresponding figure legends. Statistical significance was evaluated using one‑way or two‑way analysis of variance (ANOVA) followed by Tukey's multiple comparisons post hoc test. A p‑value < 0.05 was considered statistically significant, while "ns" indicates no significant difference. All statistical analyses were performed using Prism 8 software (GraphPad Software).

**Figures**


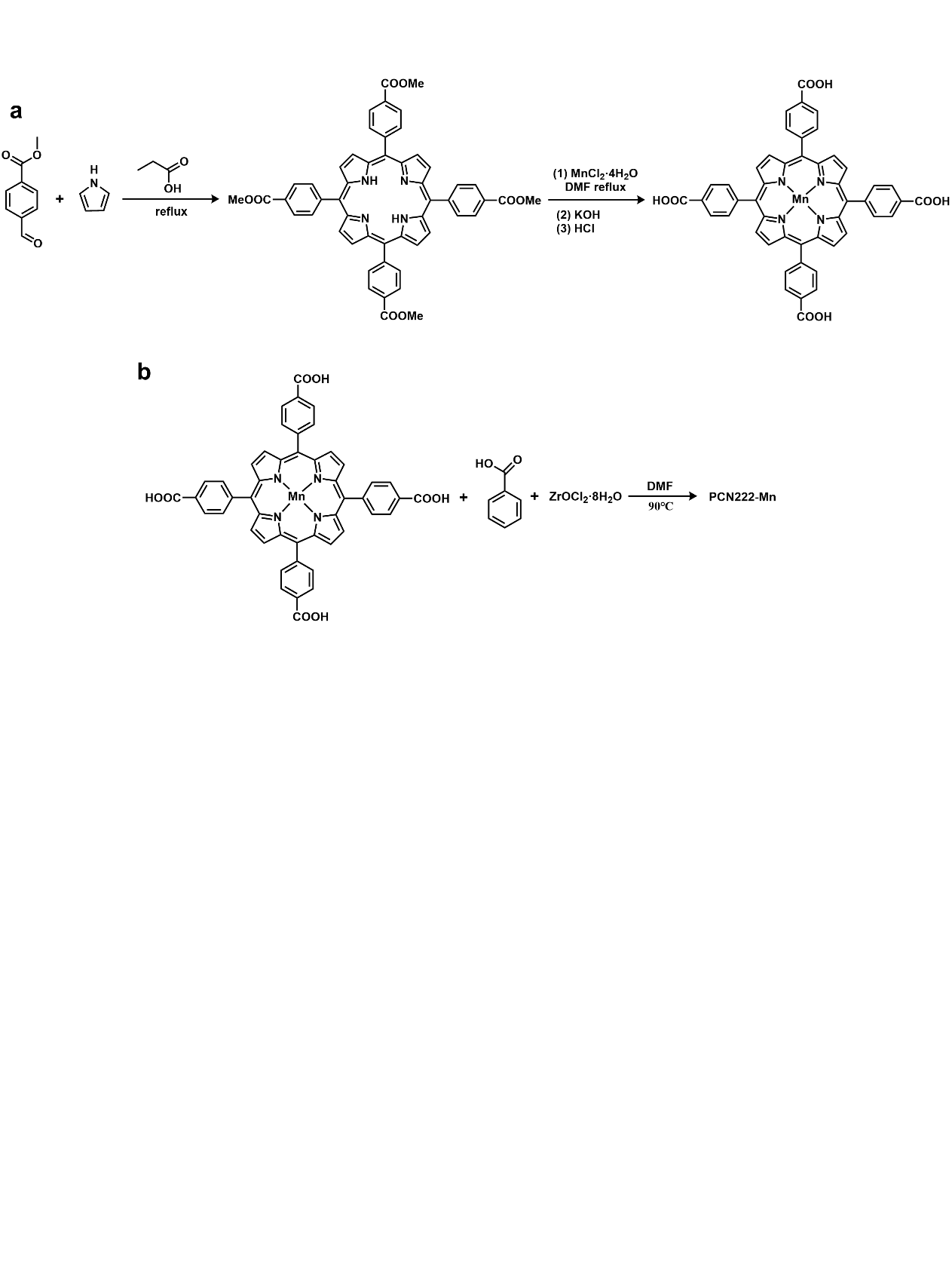


**Schem S1.** Synthetic route of a) TCPP-Mn, and b) PCN222-Mn.


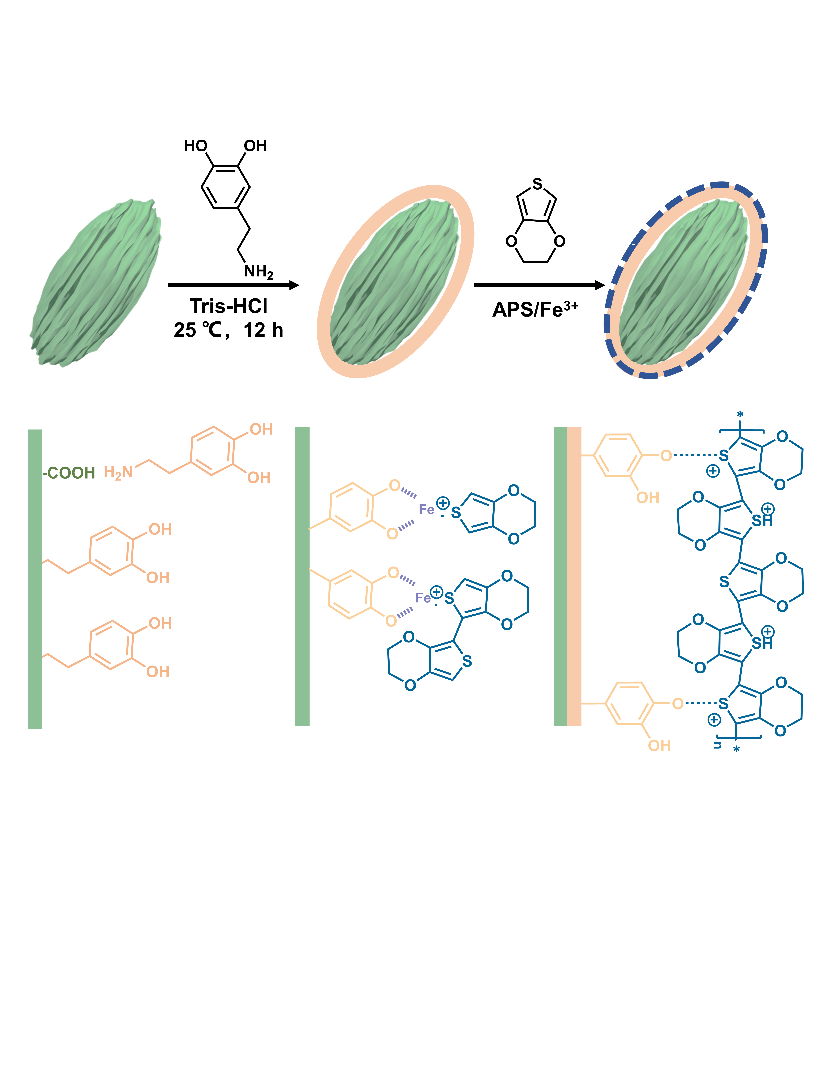


**Scheme S2.** *In situ* assembly process of the PEDOT@PMOF.


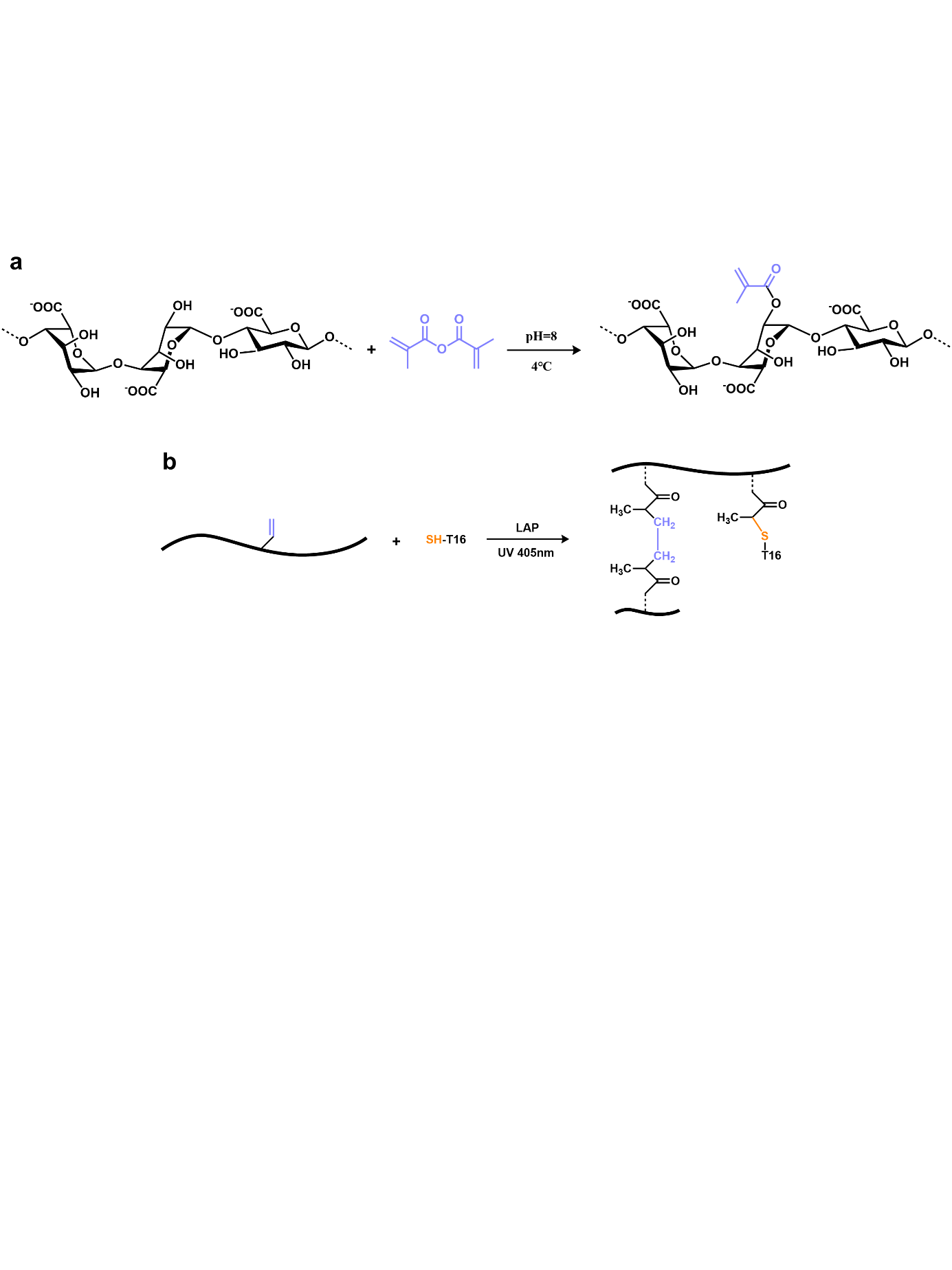


**Scheme S3.** Synthetic route and gelation process. (a) Synthetic route of AlgMA. (b) Gelation mechanism of the TAlg hydrogel.


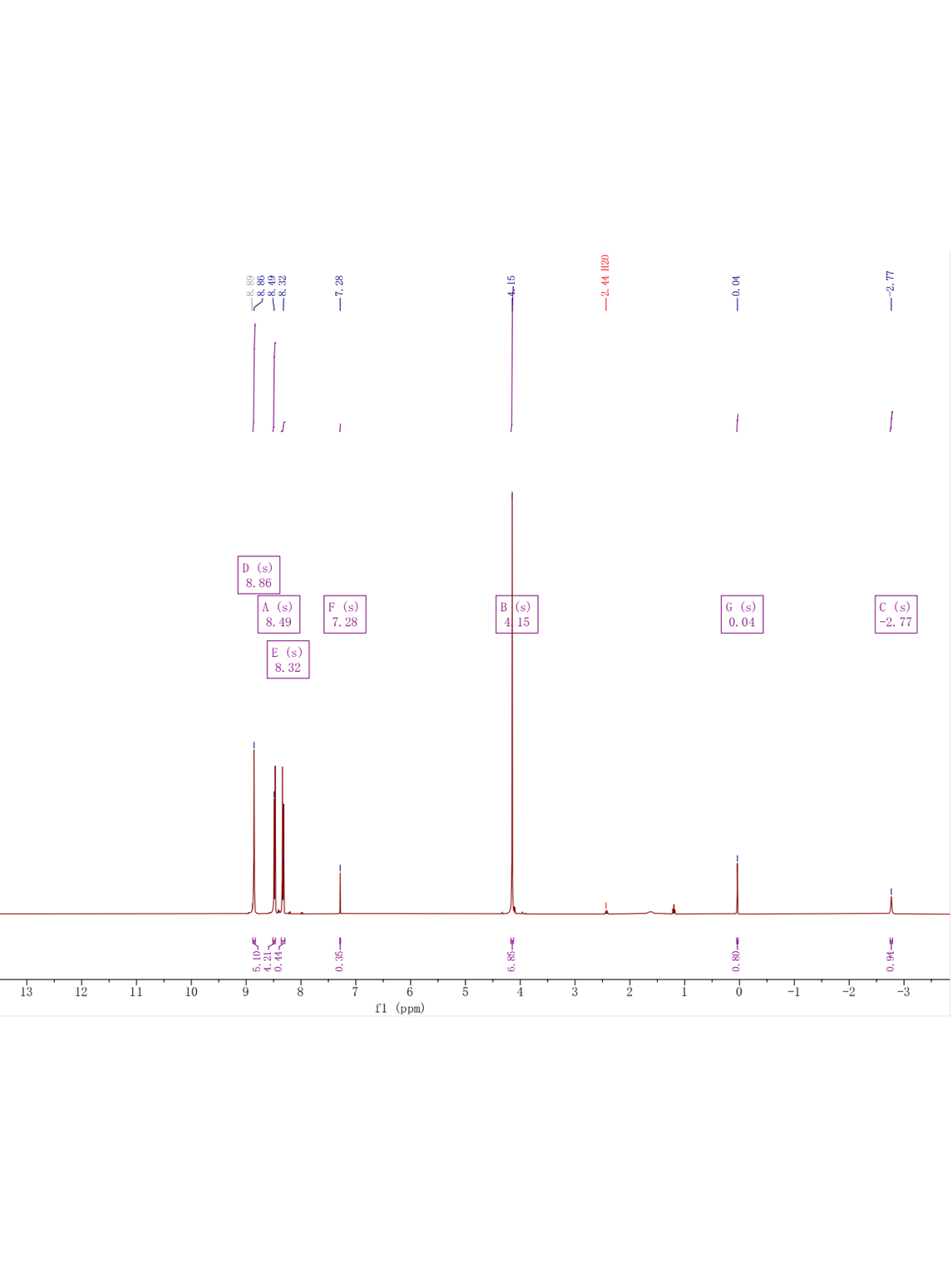


**Figure S1.** ^1^H NMR spectra of TCPP-Mn in CDCl_3_.


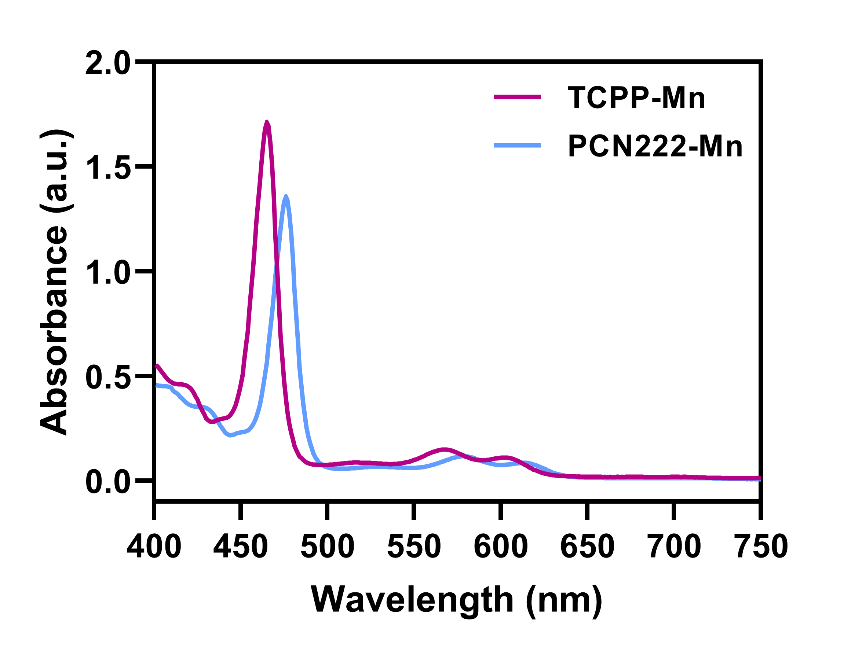


**Figure S2.** UV-Vis absorption curve of TCPP-Mn and the MOF PCN222-Mn.


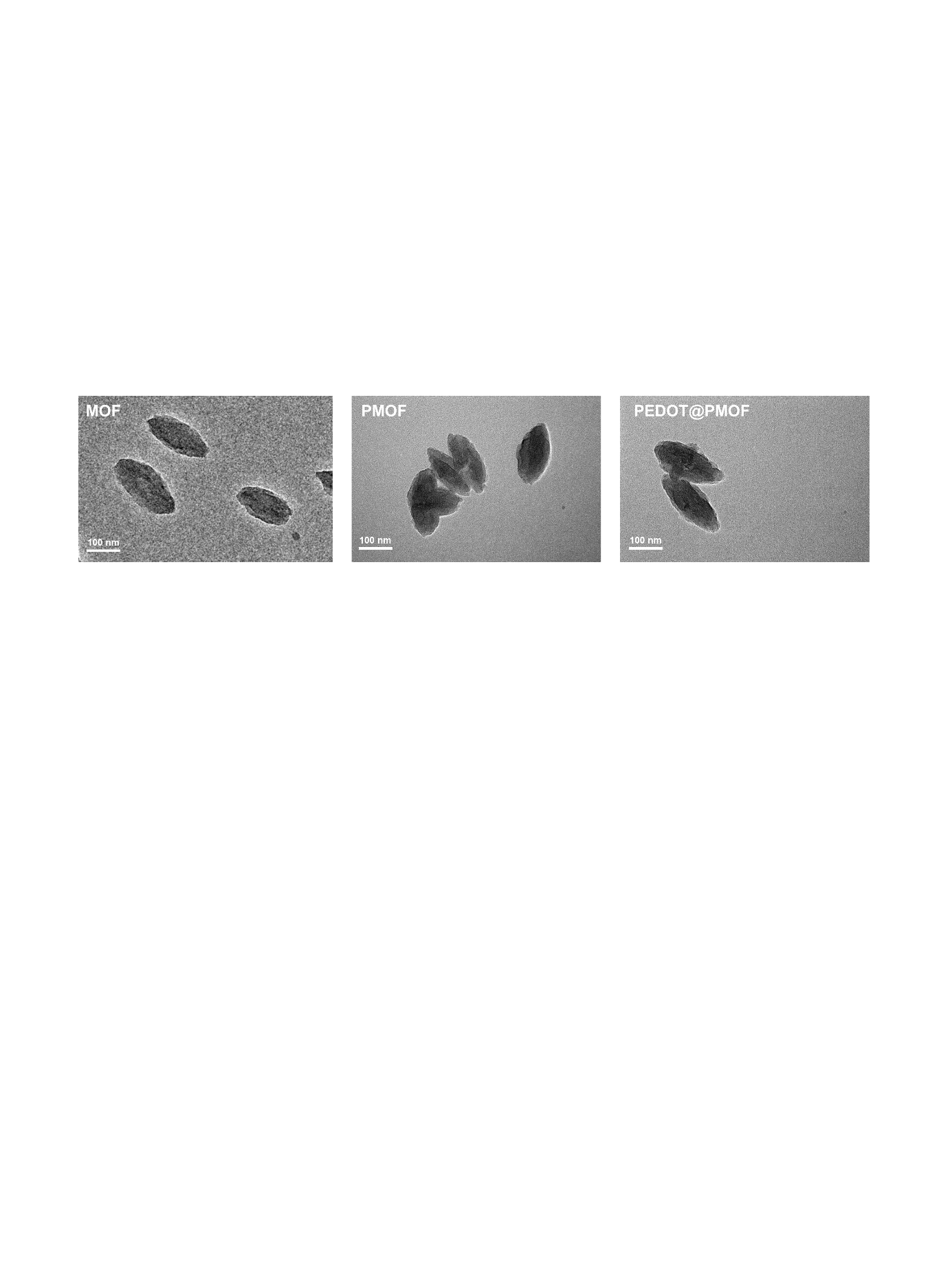


**Figure S3.** TEM image of MOF, PMOF, and PEDOT@PMOF.


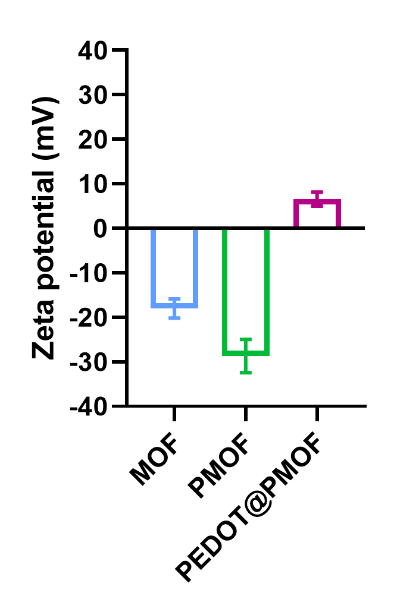


**Figure S4.** Zeta potential of MOF, PMOF, and PEDOT@PMOF. Data are presented as mean ± s.d.


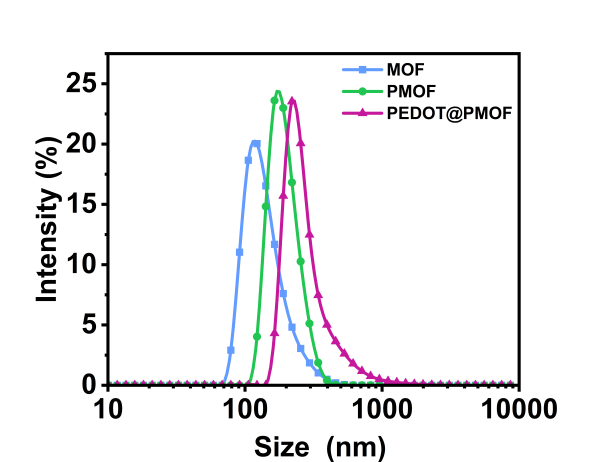


**Figure S5.** Particle size of MOF, PMOF, and PEDOT@PMOF in RO water.


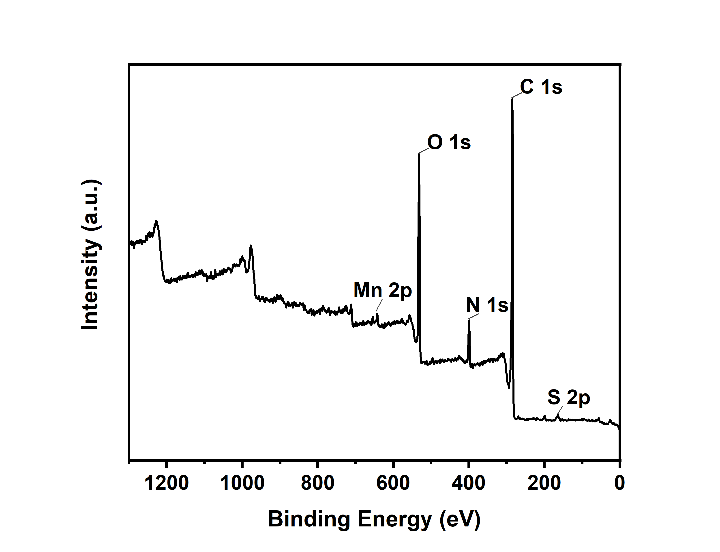


**Figure S6.** XPS survey spectrum of PEDOT@PMOF.


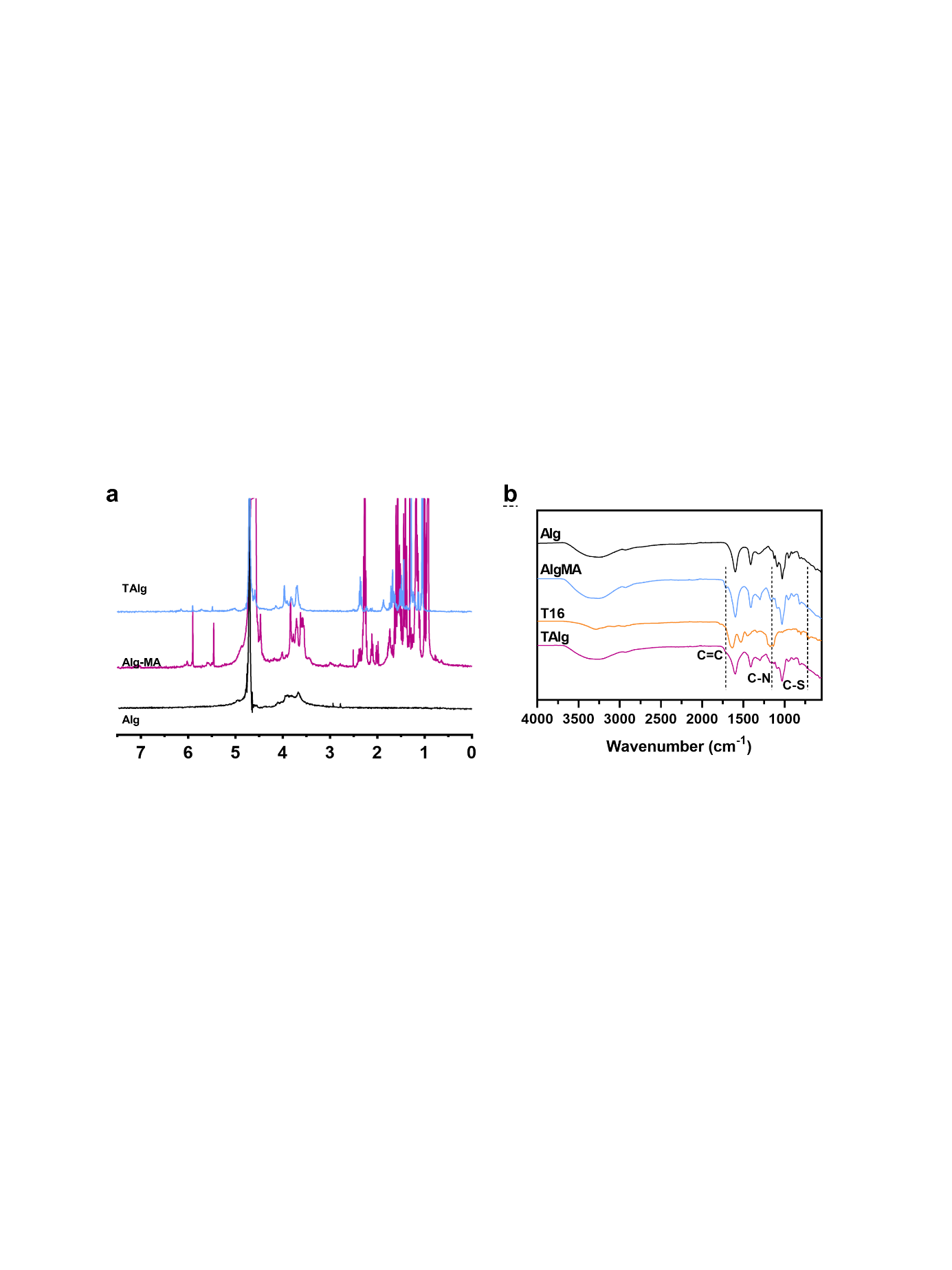


**Figure S7.** Components and structure characterization of TAlg. (a) ^1^H NMR spectra of Alg, AlgMA, and TAlg in D_2_O. (b) FT-IR spectra of Alg, AlgMA, T16 peptide, and TAlg.


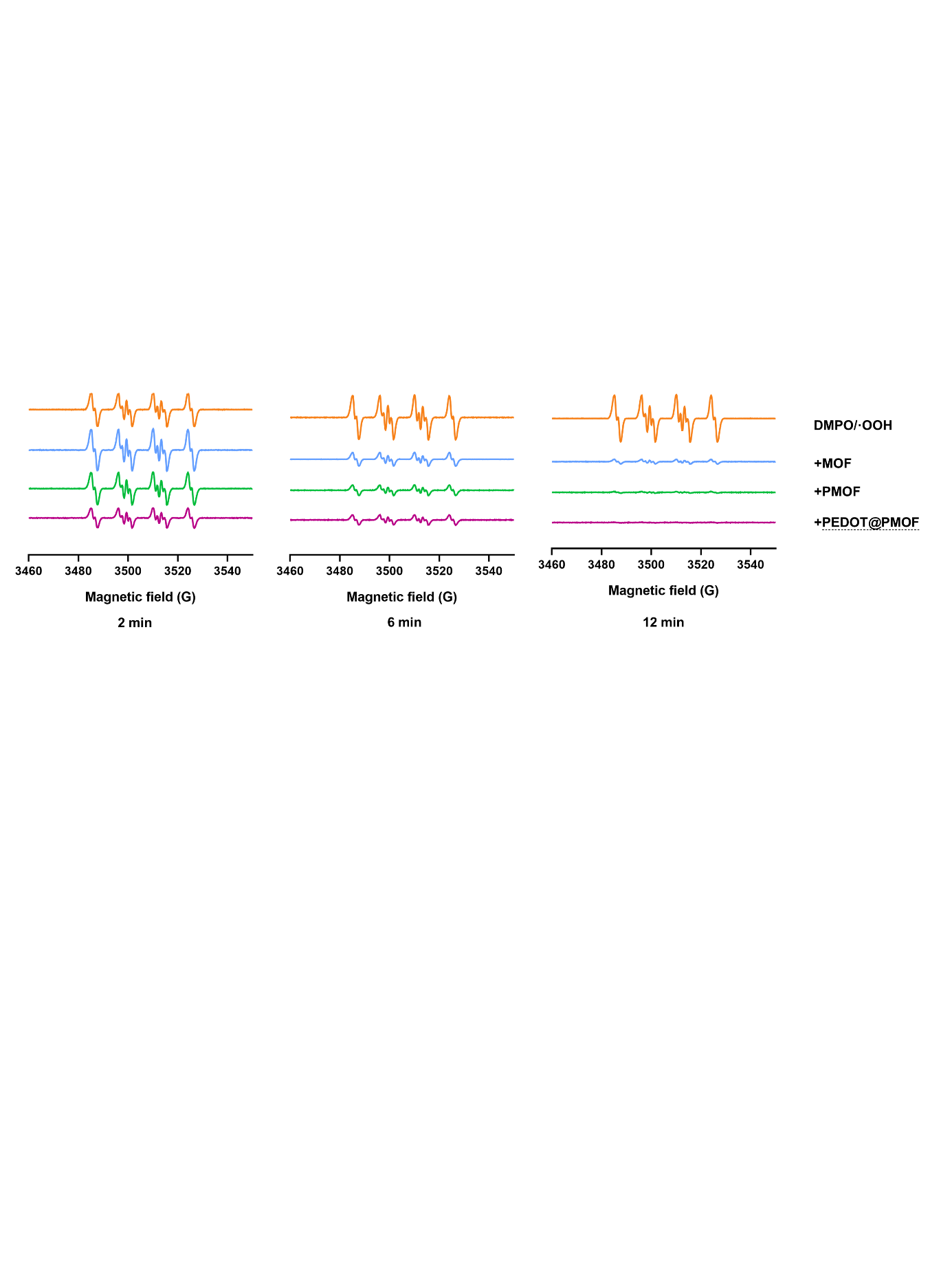


**Figure S8.** EPR for ·OOH elimination situation at 2, 6, 12 min after examination of MOF, PMOF, and PEDOT@PMOF.


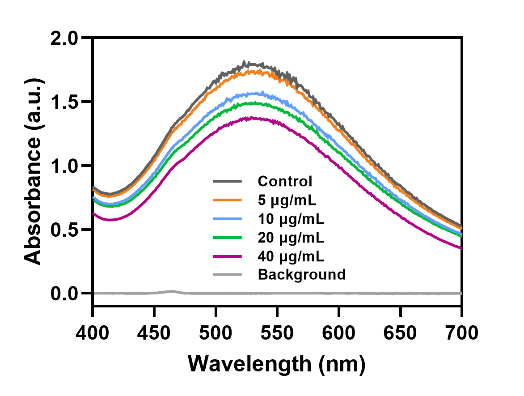


**Figure S9.** UV-Vis absorption spectra of ·OH scavenging activity of PEDOT@PMOF at different concentrations.


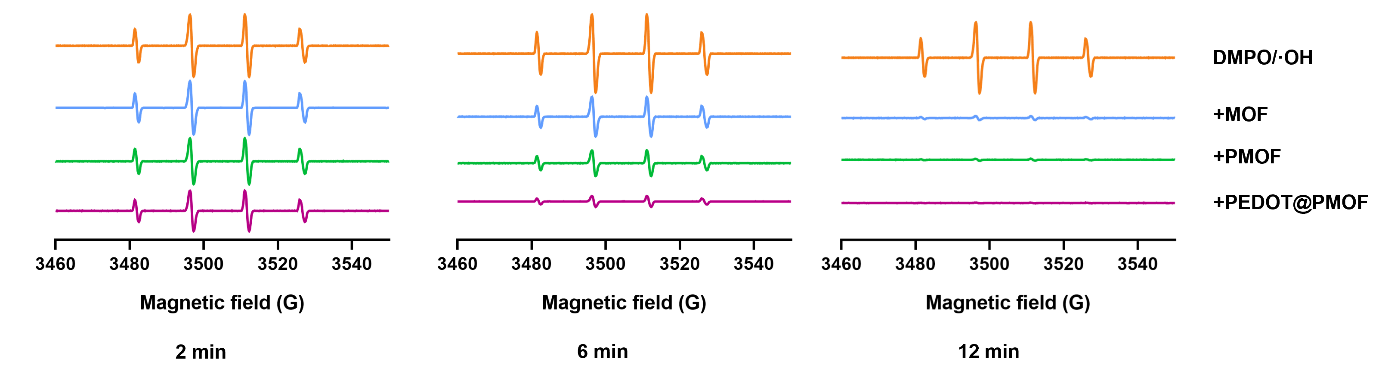


**Figure S10.** EPR for ·OH elimination situation at 2, 6, 12 min after examination of MOF, PMOF, and PEDOT@PMOF.


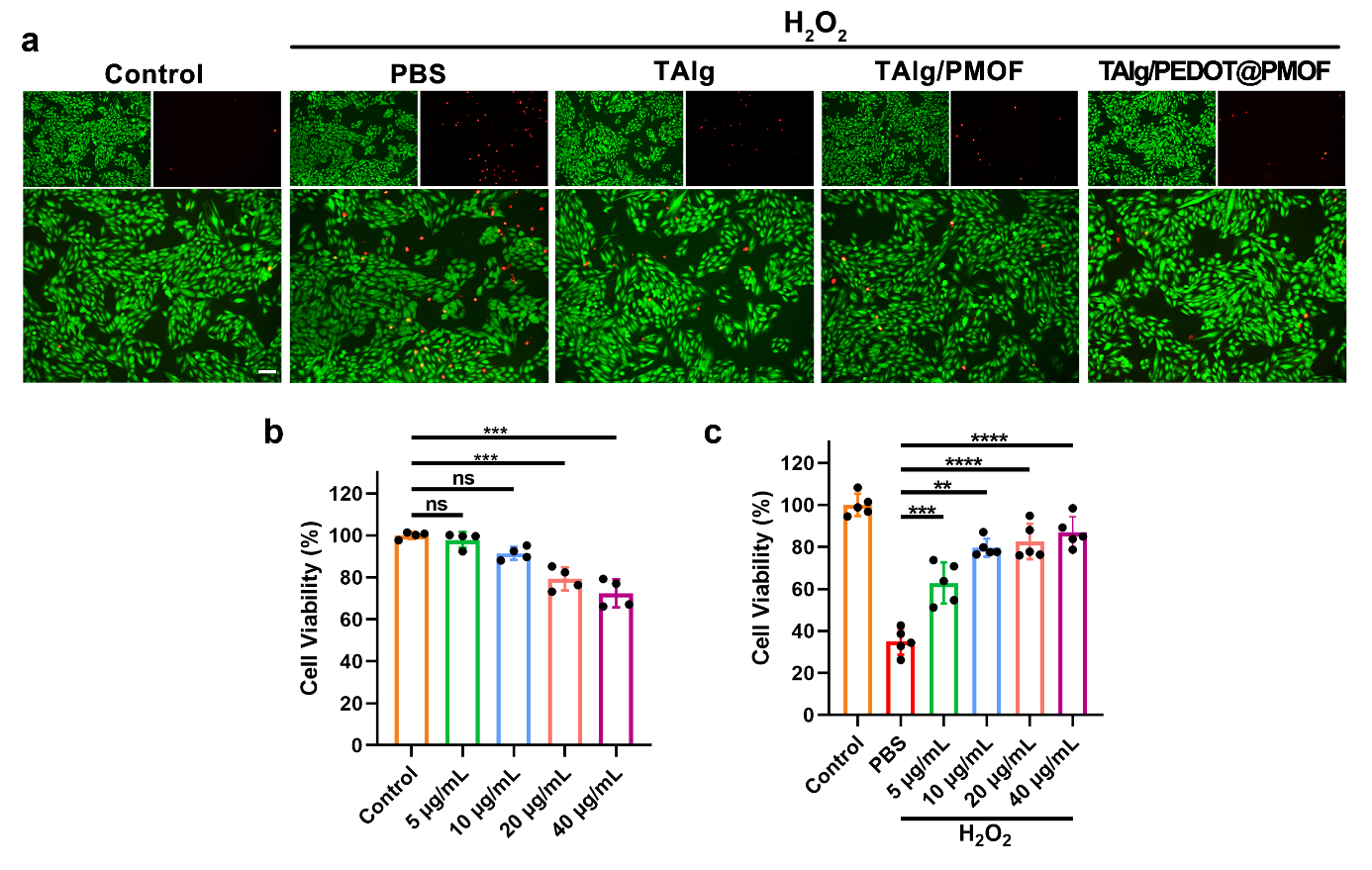


**Figure S11.** Biocompatibility and cell-protective effects of hydrogels and nanofillers. (a) The live/dead staining of H9C2 cells after being treated with H_2_O_2_ and different hydrogel samples for 24 h (Green (FDA), red (PI). Scale bar: 100 μm. (b) H9C2 cells viability treated with different concentrations of nanofillers through CCK8 assay (n=4). (c) H9C2 cells viability treated with H_2_O_2_ and different concentration of nanofillers through CCK8 assay (n=5). Data are presented as mean ± s.d., and One-way ANOVA with Tukey's multiple comparisons test was used to compare the means of the values of groups. ns indicates not significant, **p < 0.01, ***p < 0.001 and ****p <0.0001.


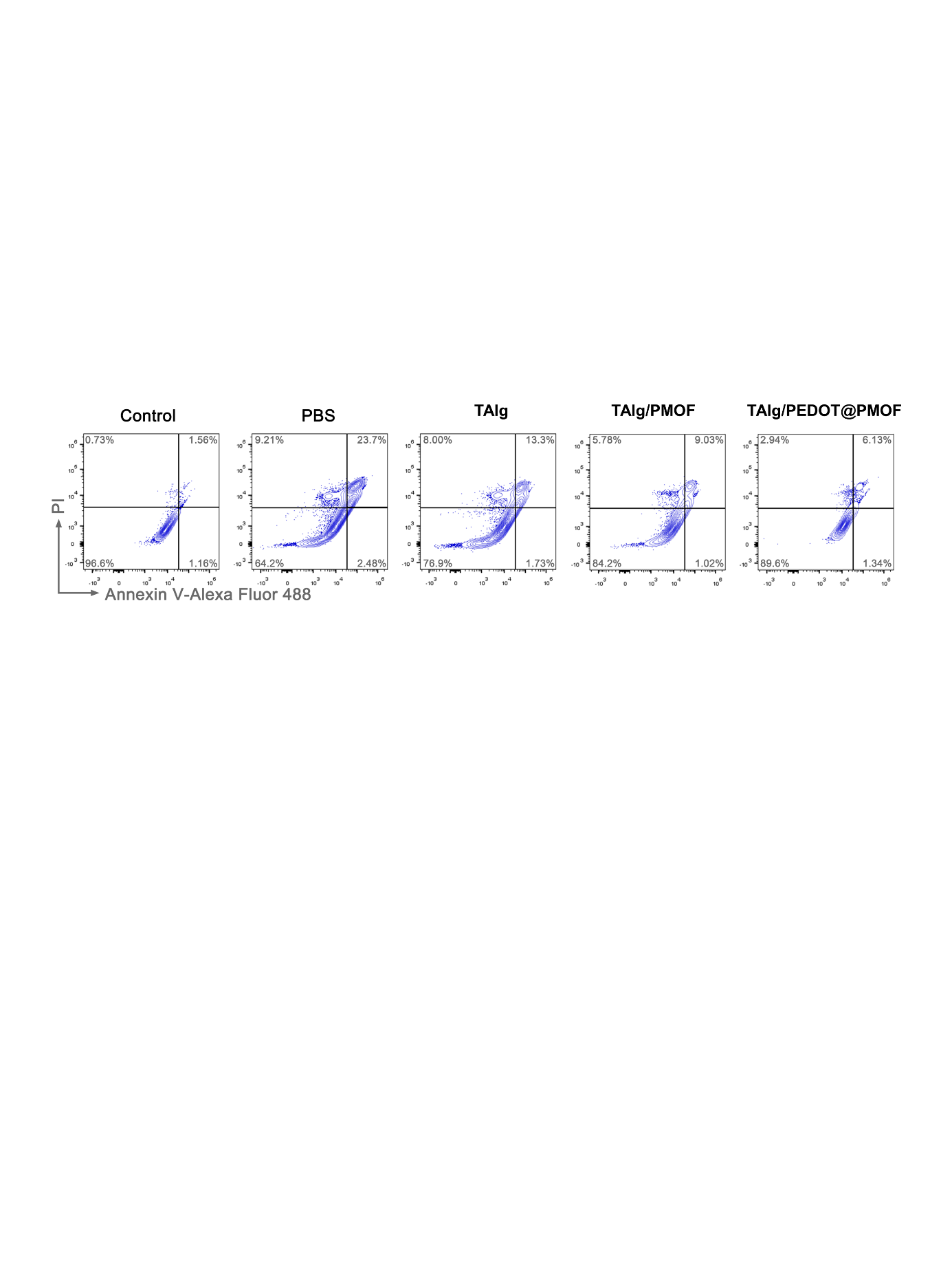


**Figure S12.** Flow cytometry results of H9C2 cells apoptosis after treatment with different hydrogel extracts.


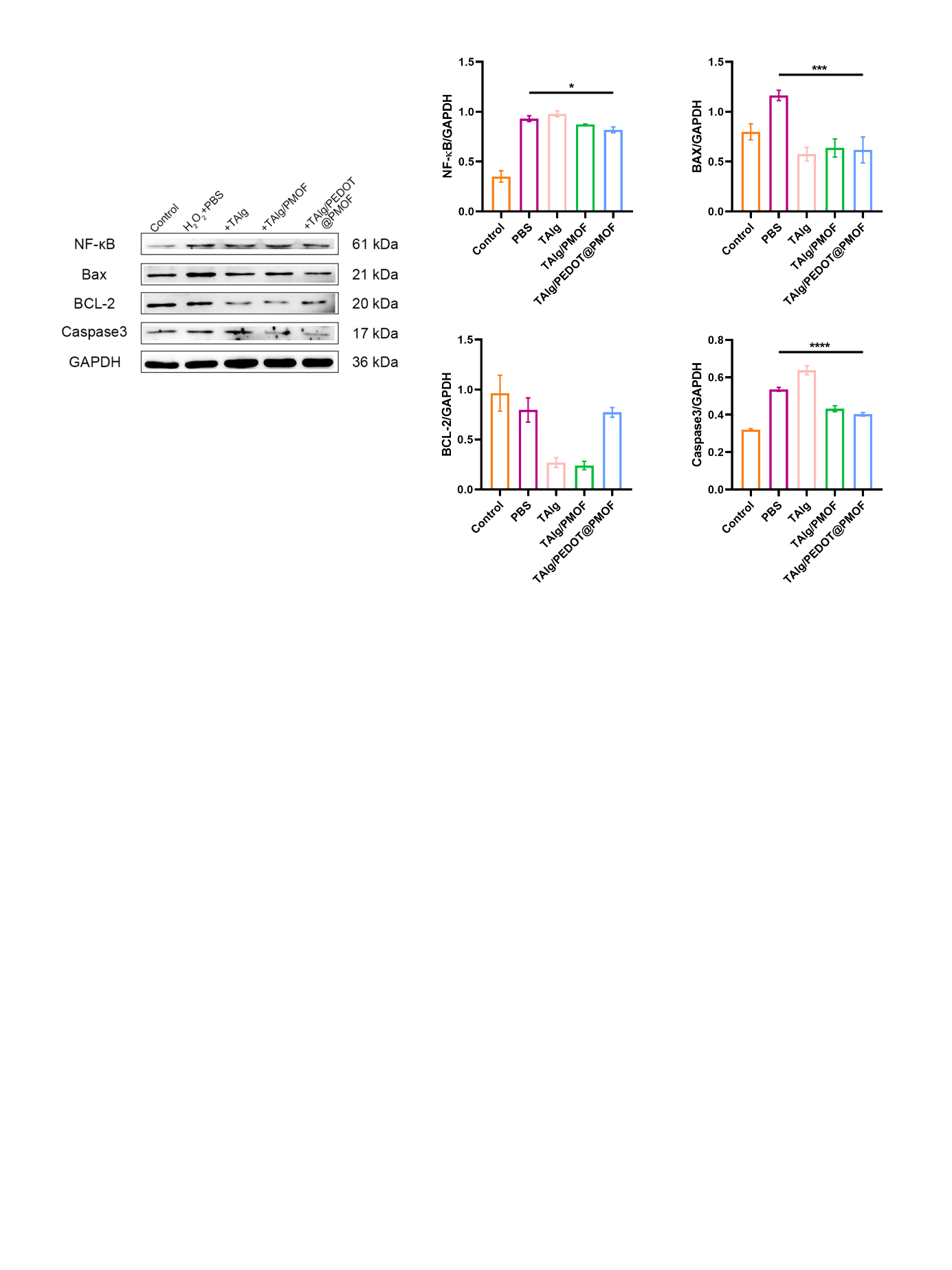


**Figure S13.** The protein expression of NF-κB, Bax, BCL-2, and Caspase3 in H9C2 cells treated with different hydrogel extracts. Data are presented as mean ± s.d., and One-way ANOVA with Tukey's multiple comparisons test was used to compare the means of the values of groups. *p < 0.5, ***p < 0.001 and ****p <0.0001.


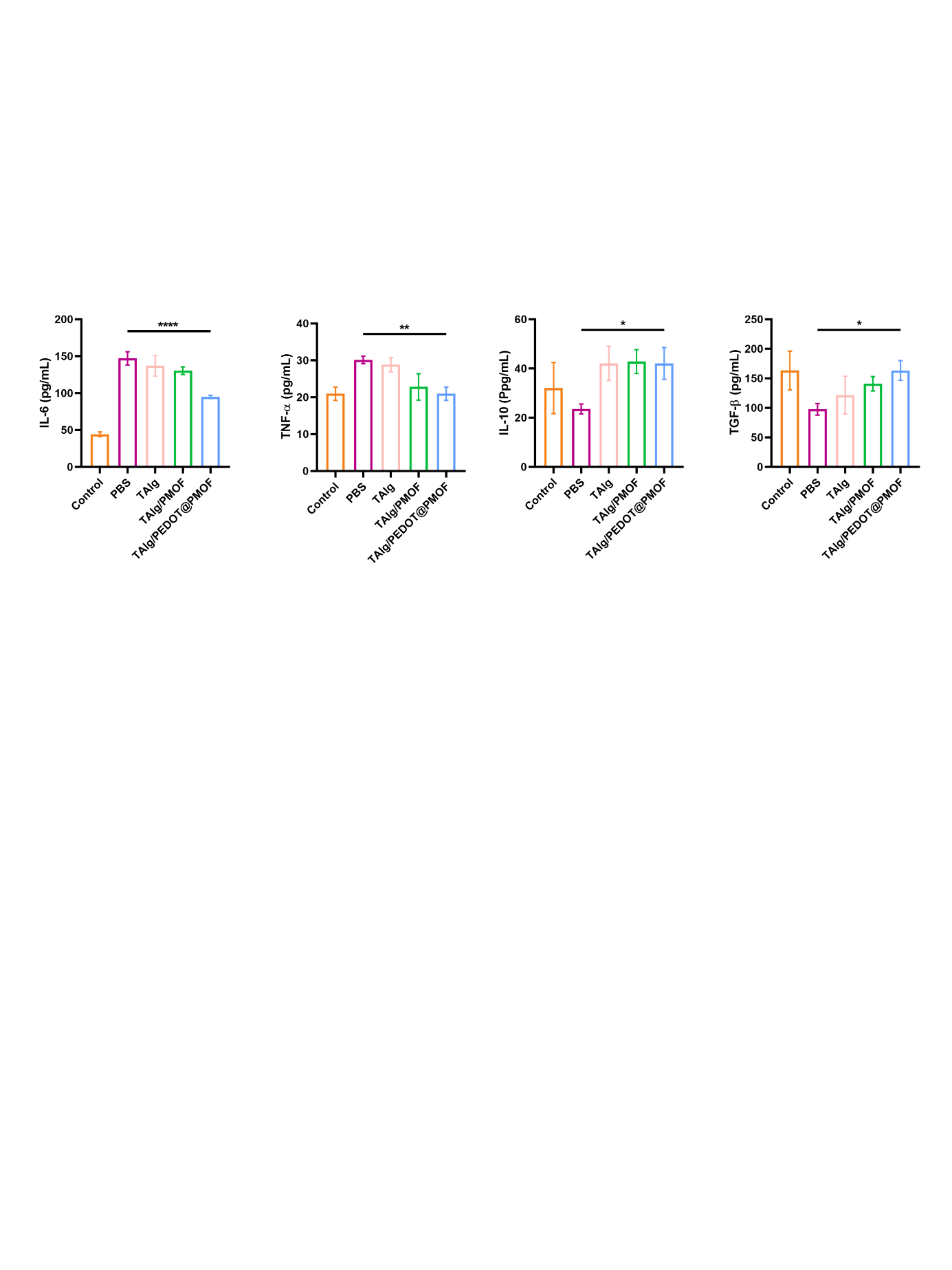


**Figure S14.** The inflammation-related cytokines of IL-6, TNF-α, IL-10, and TGF-β were measured in the supernatant of RAW264.7 cells treated with different hydrogel extracts. Data are presented as mean ± s.d., and One-way ANOVA with Tukey's multiple comparisons test was used to compare the means of the values of groups. *p < 0.5, **p < 0.01, and ****p <0.0001.


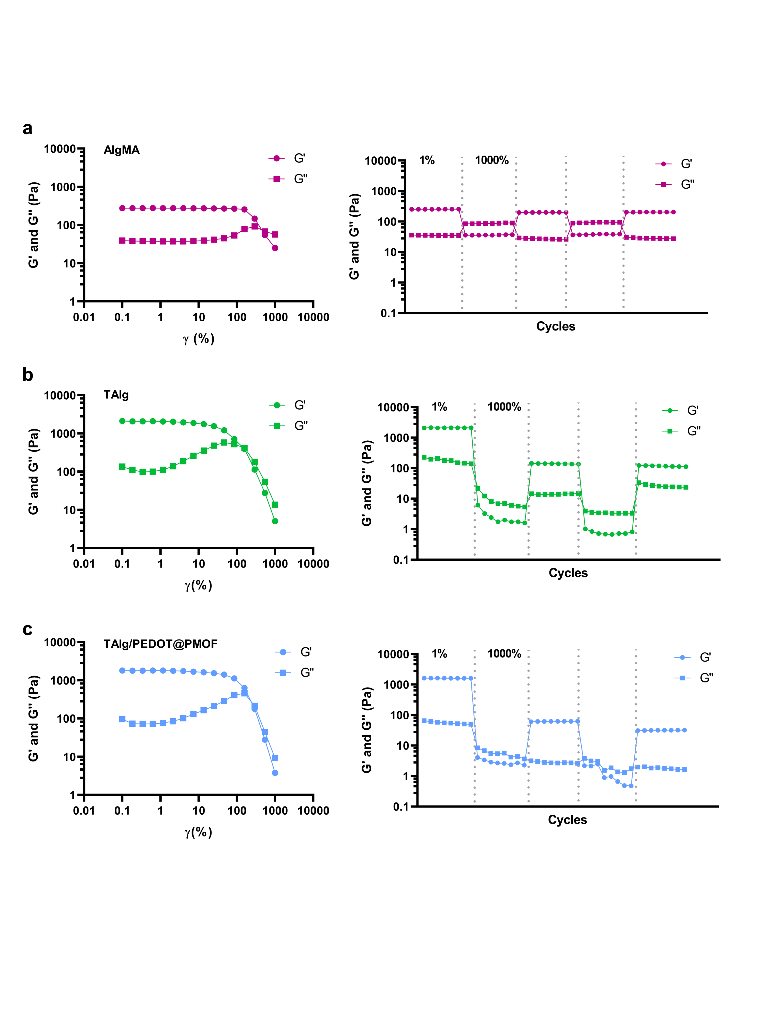


**Figure S15.** The rheological behavior of (a) AlgMA, (b) Talg, and (c) TAlg/PEDOT@PMOF hydrogels.


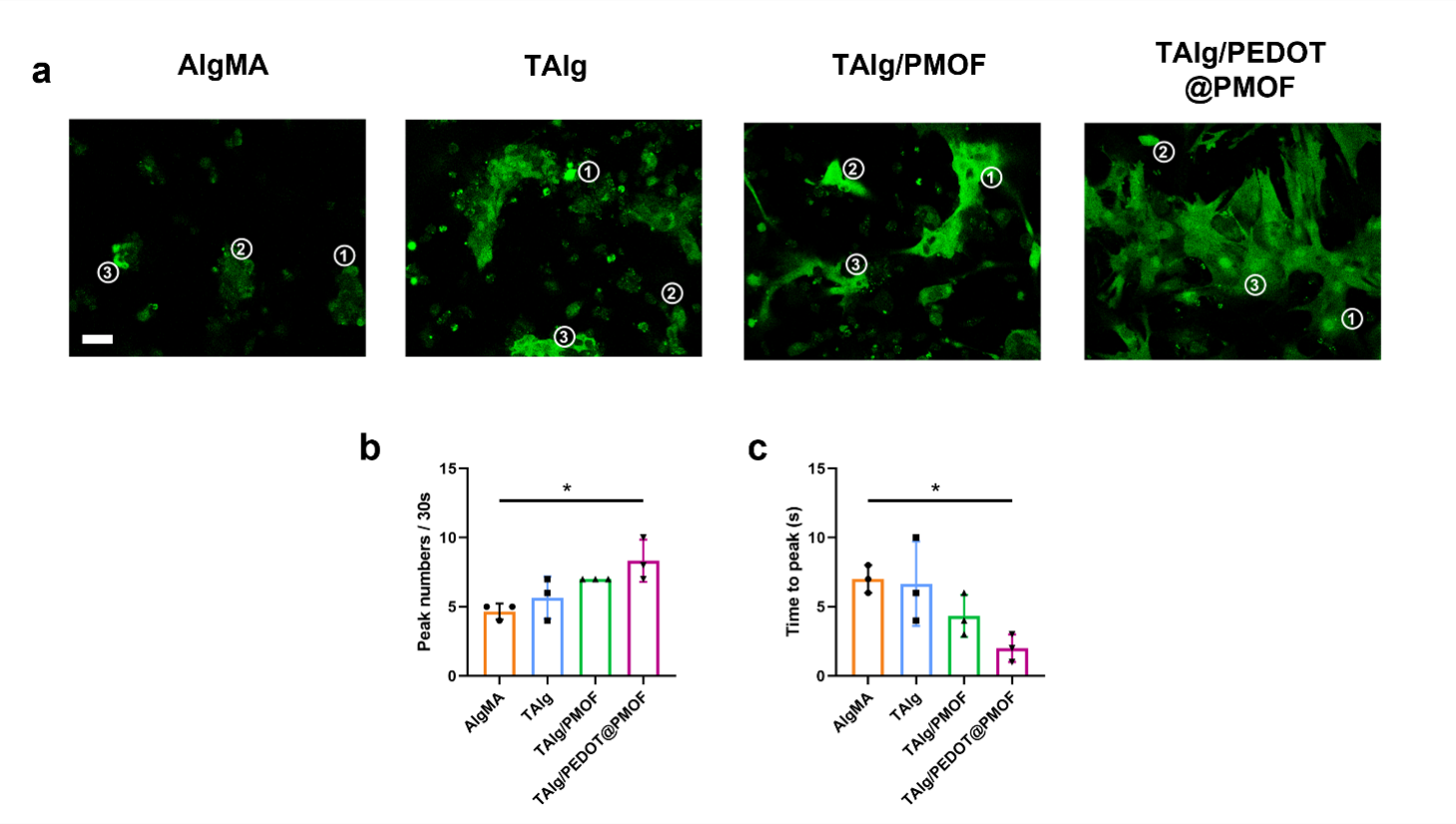


**Figure S16.** (a) Calcium transients in NRCMs in different groups. Scale bar: 40 µm. (b) The Ca^2+^ transient propagation peak (F/F0 peak) numbers. (c) Ca^2+^ transient propagation time to peak. Data are presented as mean ± s.d., and One-way ANOVA with Tukey's multiple comparisons test was used to compare the means of the values of groups. *p < 0.05.


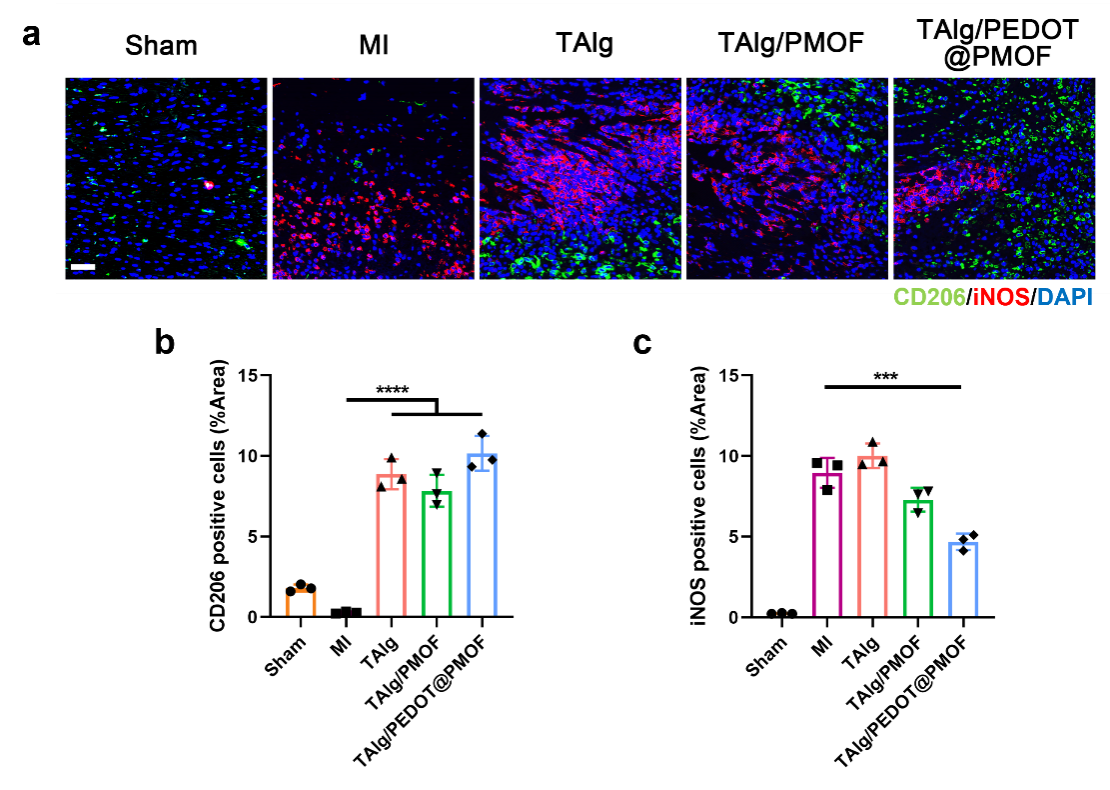


**Figure S17.** The effects of TAlg/PEDOT@PMOF on inflammation-related immune cell regulation in infarcted hearts of rats. (a) Representative images of macrophage polarization in the infarct zone as detected by immunostaining for iNOS and CD206, the M1 and M2 phenotype makers, respectively. Scale bar, 40 μm. Quantification analysis of (b) CD206 and (c) iNOS staining. Data are presented as mean ± s.d, and One-way ANOVA with Tukey's multiple comparisons test was used to compare the means of the values of groups. ***p < 0.001 and ****p <0.0001.


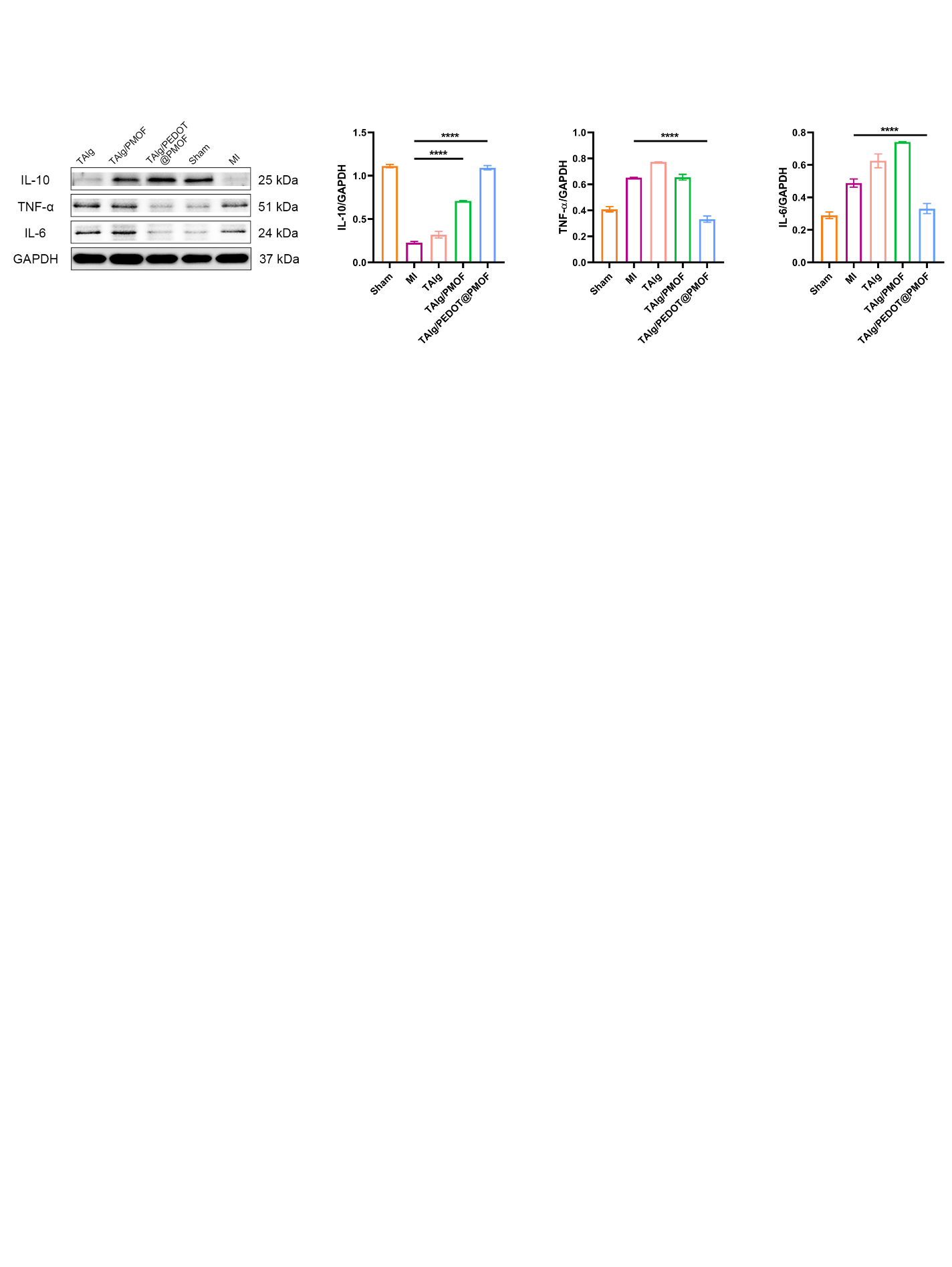


**Figure S18.** The cytokine expression of IL-10, IL-6, and TNF-α in isolated myocardial tissues treated with different hydrogels. Data are presented as mean ± s.d, and One-way ANOVA with Tukey's multiple comparisons test was used to compare the means of the values of groups, ****p < 0.0001.


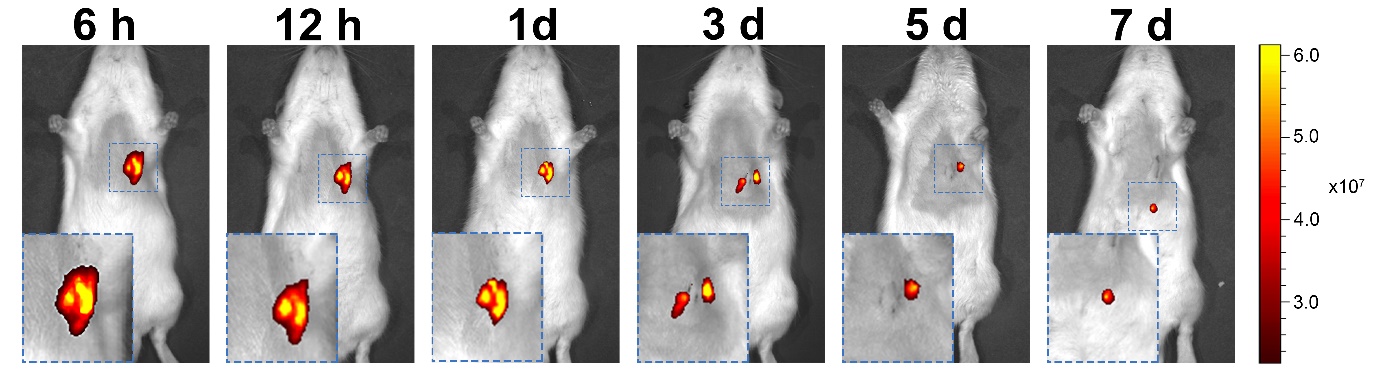


**Figure S19.** Representative fluorescence images of the rats after receiving an intramyocardial injection of TAlg/PEDOT@PMOF with ICG-labeled nanofillers for different time durations. The color bar representing the fluorescent intensity values corresponding to different colors was placed on the right.


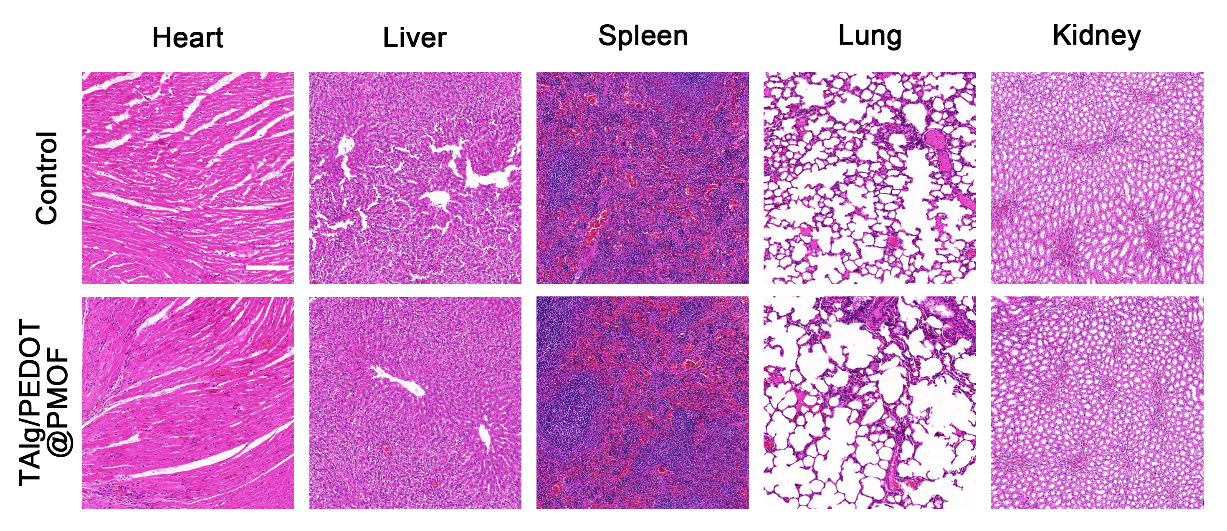


**Figure S20.** H&E-stained sections of major organs from normal rats and MI rats treated with intramyocardial injection of TAlg/PEDOT@PMOF hydrogel 48 h after injection. Scale bar, 100 μm.

**References**

[1] Y. F. Liu, Y. Cheng, H. Zhang, M. Zhou, Y. J. Yu, S. C. Lin, B. Jiang, X. Z. Zhao, L. Y. Miao, C. W. Wei, Q. Y. Liu, Y. W. Lin, Y. Du, C. J. Butch, H. Wei, *Sci. Adv.* **2020**, 6.

[2] Q. B. Xu, G. T. Zhan, Z. L. Zhang, T. Y. Yong, X. L. Yang, L. Gan, *Theranostics* **2021**, 11, 1937.

[3] D. L. Gan, Z. Q. Huang, X. Wang, D. J. Xu, S. Q. Rao, K. F. Wang, F. Z. Ren, L. L. Jiang, C. M. Xie, X. Lu, *Mater. Horiz.* **2023**, 10, 2169.

[4] A. Boddupalli, K. M. Bratlie, *Biomater. Sci.* **2019**, 7, 1188.

[5] M. Gheorghiade, C. J. Larson, S. J. Shah, S. J. Greene, J. G. F. Cleland, W. S. Colucci, P. Dunnmon, S. E. Epstein, R. J. Kim, R. V. Parsey, N. Stockbridge, J. Carr, W. Dinh, T. Krahn, F. Kramer, K. Wahlander, L. I. Deckelbaum, D. Crandall, S. Okada, M. Senni, S. Sikora, H. N. Sabbah, J. Butler, *Circ-Heart Fail* **2016**, 9.
